# Antiemesis Corticosteroids Potentiate Checkpoint Blockade Efficacy by Normalizing the Immune Microenvironment in Metastatic Murine Breast Cancer

**DOI:** 10.1002/advs.202514261

**Published:** 2025-11-28

**Authors:** John D. Martin, Koji Nagaoka, Myrofora Panagi, Akihiro Hosoi, Fotios Mpkeris, Pengwen Chen, Thahomina T. Khan, Margaret R. Martin, Changbo Sun, Chrysovalantis Voutouri, Maria Louca, Panagiotis Papageorgis, Akira Sumiyoshi, Nobuhiro Nitta, Kazuyoshi Takeda, Ichio Aoki, Kazunori Kataoka, Triantafyllos Stylianopoulos, Kazuhiro Kakimi, Horacio Cabral

**Affiliations:** ^1^ Department of Bioengineering Graduate School of Engineering The University of Tokyo Bunkyo Tokyo 113‐8656 Japan; ^2^ Department of Immunotherapeutics The University of Tokyo Hospital Bunkyo Tokyo 113‐8656 Japan; ^3^ Department of Immunology School of Medicine Kindai University Osakasayama Osaka 589‐8511 Japan; ^4^ Cancer Biophysics Laboratory Department of Mechanical and Manufacturing Engineering University of Cyprus Nicosia 1678 Cyprus; ^5^ Cancer Genetics, Therapeutics & Ultrastructural Pathology Department The Cyprus Institute of Neurology & Genetics Nicosia 2371 Cyprus; ^6^ National Institute of Radiological Sciences Japan Agency for Quantum and Radiological Science and Technology Anagawa 4‐9‐1, Inage Chiba 263‐8555 Japan; ^7^ Department of Life Sciences Program in Biological Sciences European University Cyprus Nicosia 1516 Cyprus; ^8^ Division of Cell Biology Biomedical Research Center Juntendo University Bunkyo Tokyo 113‐8421 Japan; ^9^ Department of Biofunctional Microbiota Graduate School of Medicine Juntendo University Bunkyo Tokyo 113‐8421 Japan; ^10^ Innovation Center of NanoMedicine Kawasaki Institute of Industrial Promotion Kawasaki Kanagawa 210‐0821 Japan

**Keywords:** 3D magnetic resonance (MR) angiography, anti‐PD‐1 and anti‐CTLA‐4 antibodies, breast cancer, dexamethasone, tumor normalization, tumor perfusion

## Abstract

Glucocorticoid steroids are widely prescribed in oncology for managing treatment‐related side effects, though their use is discouraged in patients undergoing immune checkpoint blockade (ICB). Given the crucial role of steroids in supportive care, it is imperative to develop integrated strategies that do not compromise ICB therapy effectiveness. Here, protocols of dexamethasone resembling clinical antiemesis regimens are tested in murine models of ICB‐resistant breast cancer. Unexpectedly, dexamethasone enhanced the efficacy of ICB in models of primary cancer and spontaneous metastasis. Dexamethasone increased the volume of blood vessels within tumors, thereby facilitating lymphocyte infiltration and intratumor distribution. Additionally, dexamethasone depleted immunosuppressive cells and potentiated the capacity of ICB to enrich tumors of antigen‐recognizing cytotoxic lymphocytes. Importantly, it is found that dexamethasone does not abolish the functions of antigen‐naïve lymphocytes and has no effect on the activity of antigen‐experienced lymphocytes. These findings align with recent clinical guidelines and meta‐analyses, suggesting that dexamethasone deserves further exploration in ICB protocols to potentially benefit efficacy. Our study supports the notion that the current practice of avoiding short‐term glucocorticoid steroids in patients receiving ICB should be reconsidered toward enabling the use of steroids in clinical trials of new drug combinations.

## Graphical abstract

Corticosteroids improve the efficacy of immune checkpoint blockade therapy in metastatic murine breast cancer. By normalizing the tumor immune microenvironment, corticosteroids reduce immunosuppressive signals, restore T‐cell function, and promote antitumor immune responses, resulting in enhanced tumor control.

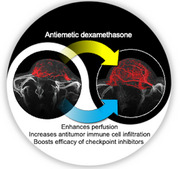

## 1 Introduction

Immune checkpoint blockade (ICB) is revolutionizing cancer therapy, but resistance limits its benefit to only a minority of patients.^[^ ^1^ ^]^ Mechanisms of resistance to ICB involve immunosuppression in the tumor microenvironment (TME).^[^ ^2^ ^]^ Accordingly, systemic and local immunosuppression caused by coadministration of glucocorticoid steroids worsens outcomes with ICB.^[^ ^3^, ^4^, ^5^ ^]^ Consequently, less than half of clinical trials evaluating ICB allow steroids.^[^ ^6^ ^]^ Nonetheless, steroids are important in oncology,^[^ ^5^ ^]^ as they are used to manage immune‐related adverse events caused by ICB,^[^ ^7^ ^]^ cancer‐related symptoms (e.g., edema),^[^ ^4^ ^]^ and underlying co‐morbidities (e.g., autoimmune conditions and organ transplant).^[^ ^3^ ^]^ Steroids are also used for prophylaxis of adverse events (e.g., hypersensitivity and emesis) caused by certain chemotherapies^[^ ^6^, ^8^ ^]^ and potentially even for ICB.^[^ ^9^ ^]^ First‐line, triple‐negative breast cancer is one treatment setting where ICB is only administered in combination with chemotherapies that sometimes require pretreatment steroids. Nonetheless, the specific effects of antiemetic dexamethasone on the immune microenvironment and ICB response in breast cancer are not yet described.^[^ ^6^ ^]^

Dexamethasone administered for emesis prophylaxis in chemo‐immunotherapy regimens begins before ICB administration and continues for several days. An American Society of Clinical Oncology expert panel recommended continuing the use of steroids in this setting.^[^ ^10^ ^]^ Although some researchers found this unexpected,^[^ ^6^ ^]^ the expert panel's recommendation was based on two pertinent studies showing that adding ICB to chemotherapy and antiemetic steroids resulted in higher survival compared to chemotherapy and steroids alone.^[^ ^11^, ^12^ ^]^ In other words, the studies highlighted by the expert panel found that steroids did not negate the benefits of ICB.^[^ ^11^, ^12^ ^]^ Moreover, a subsequent meta‐analysis unexpectedly found that antiemetic steroids appear to improve survival in patients receiving ICB and chemotherapy.^[^ ^13^ ^]^ Nevertheless, whether antiemesis steroids enhance chemo‐immunotherapy efficacy in patients remains uncertain, as there is a paucity of data on how these antiemetic regimens impact ICB effectiveness and alter intratumor immunosuppression.^[^ ^6^ ^]^ Some clinical studies indicated that pretreatment with dexamethasone could reduce ICB‐induced adverse events leading to longer treatment adherence.^[^ ^9^ ^]^ One recent preclinical study determined that dexamethasone immediately preceding ICB did not compromise antitumor efficacy.^[^ ^14^ ^]^ How a dexamethasone protocol before and during ICB treatment affects efficacy remains unclear.

We previously demonstrated in murine breast cancer that a regimen of dexamethasone modeled after a clinical antiemesis protocol (NCT02043288)^[^ ^8^ ^]^ reverses angiogenesis, fibrosis and hypoxia signaling.^[^ ^15^ ^]^ Shorter duration and lower doses did not affect these processes, while higher doses depleted tumor blood vessels, thereby exacerbating hypoxia.^[^ ^15^ ^]^ Accordingly, only the antiemesis dexamethasone regimen remodeled tumor blood vessels to a normal, efficient phenotype fortified with perivascular cells^[^ ^15^, ^16^, ^17^, ^18^ ^]^ and reduced hyaluronan production, which reversed vessel compression.^[^ ^15^, ^19^, ^20^ ^]^ Given that fortified and decompressed vessels have increased perfusion and oxygen delivery,^[^ ^15^, ^21^, ^22^ ^]^ and that restoring oxygen delivery to tumors can alleviate immunosuppression and enhance the efficacy of ICB,^[^ ^23^, ^24^, ^25^ ^]^ we sought to test the hypothesis that this antiemesis dexamethasone protocol can increase ICB efficacy by alleviating immunosuppression in the TME.

As a preliminary step toward the potential use of a dexamethasone pretreatment to ICB for prophylactic emesis and ICB‐induced adverse events, this study aims to verify whether antiemesis dexamethasone could enhance the antitumor efficacy of ICB in murine breast cancer models.

## 2 Results

### 2.1 Dexamethasone Potentiates ICB Efficacy in Primary and Spontaneous Models of Breast Cancer

**Figure 1:**
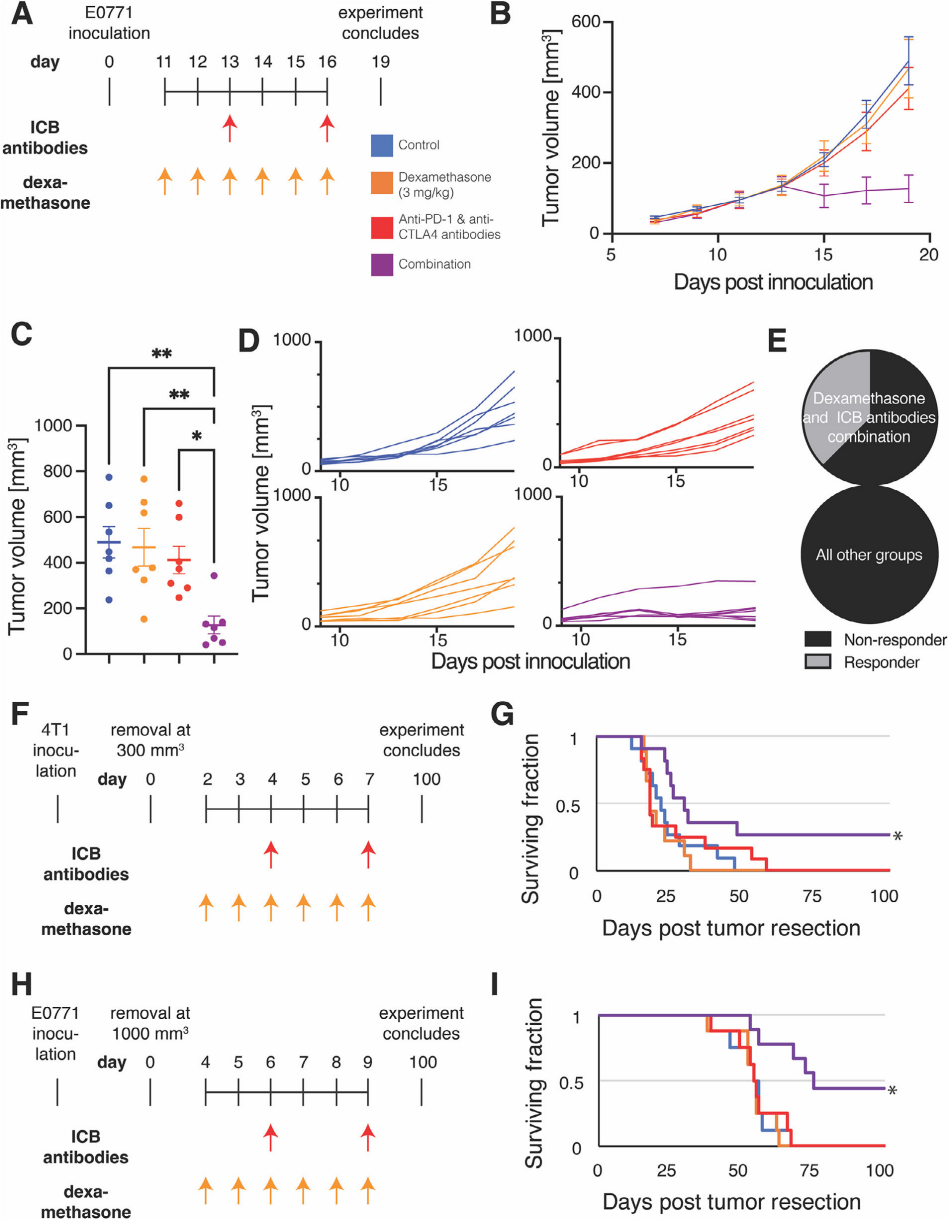
Dexamethasone potentiates an ICB cocktail in primary tumors and spontaneous metastasis models of breast cancer. A) Experimental scheme for experiment testing efficacy in E0771 primary tumors. The mice were treated with dexamethasone daily from day 11 until day 16 and immune checkpoint blockade (ICB) cocktail on days 13 and 16. The experiment was terminated on day 19. B) Graph of tumor volume over time throughout the experiment. *n* = 7 mice per group. Data plotted as average +/‐ standard error of the mean. C) Graph of tumor volume on day 19. Statistical test by one‐way ANOVA with Tukey's correction ** denotes *P*<0.01, and * denotes *P* < 0.05 (*n* = 7). D) Graphs of the tumor volume for individual mice over time throughout the experiment. E) Fraction of mice responding to treatment. Pie graphs indicate 3 responder (gray) and 5 non‐responder mice (black) in the combination group featuring dexamethasone and ICB cocktail. The other groups do not have responders. F) Experimental scheme for experiment testing efficacy in the 4T1 spontaneous metastasis model. The mice were inoculated with 4T1 cells, and their tumors were removed when they reached 300 mm^3^. The mice were treated with dexamethasone daily from day 2 until day 7 and ICB cocktail on days 4 and 7. G) Kaplan‐Meier survival curves of mice bearing spontaneous 4T1 metastases arising from surgically removed primary tumors. After recovery, mice were treated with control (blue), dexamethasone monotherapy (orange), ICB cocktail of anti‐PD‐1 and anti‐CTLA‐4 antibodies (red) or the combination (purple). *n* = 9–12 mice per group. H) Experimental scheme for experiment testing efficacy in the E0771 spontaneous metastasis model. The mice were treated with dexamethasone daily from day 4 until day 9 and ICB cocktail on days 6 and 9. I) Kaplan‐Meier survival curves of mice bearing spontaneous E0771 breast cancer metastases arising from surgically removed primary tumors. n = 8 mice per group. For Kaplan‐Meier survival curves, ^*^ *P*<0.05, log‐rank test with Holm‐Bonferroni's correction for multiple comparisons.

We first investigated the effects on ICB efficacy of an antiemesis protocol (Table S1, Supporting Information) designed such that ICB would be administered during the window of reversed angiogenesis, fibrosis, and hypoxia that we previously observed.^[^ ^15^ ^]^ We inoculated mice with E0771 breast tumors and initiated a combined treatment regimen 11 days later (**Figure** **1**A). While measuring tumor volume over time (Figure 1B), we found that neither dexamethasone monotherapy nor an ICB cocktail of anti‐PD‐1 and anti‐CTLA‐4 antibodies induced a tumor growth delay, but the combination did (Figure 1C). Such an ICB cocktail is approved for melanoma and renal cell carcinoma patients, but murine breast cancer models are often resistant to it.^[^ ^26^, ^27^, ^28^ ^]^ Inspecting the tumor volume profiles for individual tumors (Figure 1D), we determined that only in the combination group did some tumors become smaller compared to their pretreatment volumes, with three out of eight mice responding (Figure 1E).

Because the trial of atezolizumab in metastatic triple‐negative breast cancer used a nanoparticle formulation of paclitaxel in part to avoid glucocorticoid steroid use,^[^ ^29^ ^]^ we next tested the effect of dexamethasone on the efficacy of the ICB cocktail in the metastatic setting. We implanted 4T1 and E0771 as primary tumors and resected the tumors once they reached a size sufficient to induce metastasis.^[^ ^26^, ^27^, ^28^ ^]^ We then waited 2 days or 4 days for 4T1 or E0771, respectively, before initiating treatment (Figure 1F). 4T1 is resistant to this ICB cocktail,^[^ ^24^, ^25^, ^26^, ^28^ ^]^ but we found that the combination with dexamethasone significantly increased survival compared to ICB alone (Figure 1G) with a 27% cure rate and a 37% increase in median survival compared to controls (30 days vs. 22 days; P = 0.02). The body weight of mice given the combination treatment did not change, indicating it was tolerated (Figure S1A, Supporting Information). The three surviving mice were re‐implanted with 1 mm^3^ tumor chunks as a re‐challenge and compared to three age‐matched control mice (Figure S1B, Supporting Information). Tumors grew in all three control mice, but only in one previously treated, surviving mouse (Figure S1C, Supporting Information). Lungs extracted from the two remaining surviving mice did not have macroscopic metastasis (Figure S2, Supporting Information). As in studies of 4T1, previous reports indicate that metastases of E0771 have little^[^ ^27^, ^30^ ^]^ or no sensitivity to this ICB cocktail^[^ ^26^, ^28^, ^31^ ^]^ when treated with a similar protocol (Figure 1H). We found that the combination with dexamethasone significantly increased survival compared to ICB alone (Figure 1I) with a 38% cure rate and a 34% increase in median survival compared to controls (73.5 days vs. 55 days; P < 0.01). The three surviving mice were re‐inoculated with cancer cells as a re‐challenge and compared to three age‐matched control mice (Figure S1D, Supporting Information). In all three control mice, tumors grew, while in the rechallenge cohort, two mice remained tumor free, and the third mouse died tumor free one week after inoculation (Figure S1E, Supporting Information). These results indicate that dexamethasone potentiates the efficacy of an ICB cocktail against primary breast tumors and spontaneous models of metastasis.

### 2.2 Dexamethasone Promotes ICB‐Induced Enhancement of Antitumor Lymphocytes While Alleviating Expansion of Immunosuppressive Cells

**Figure 2:**
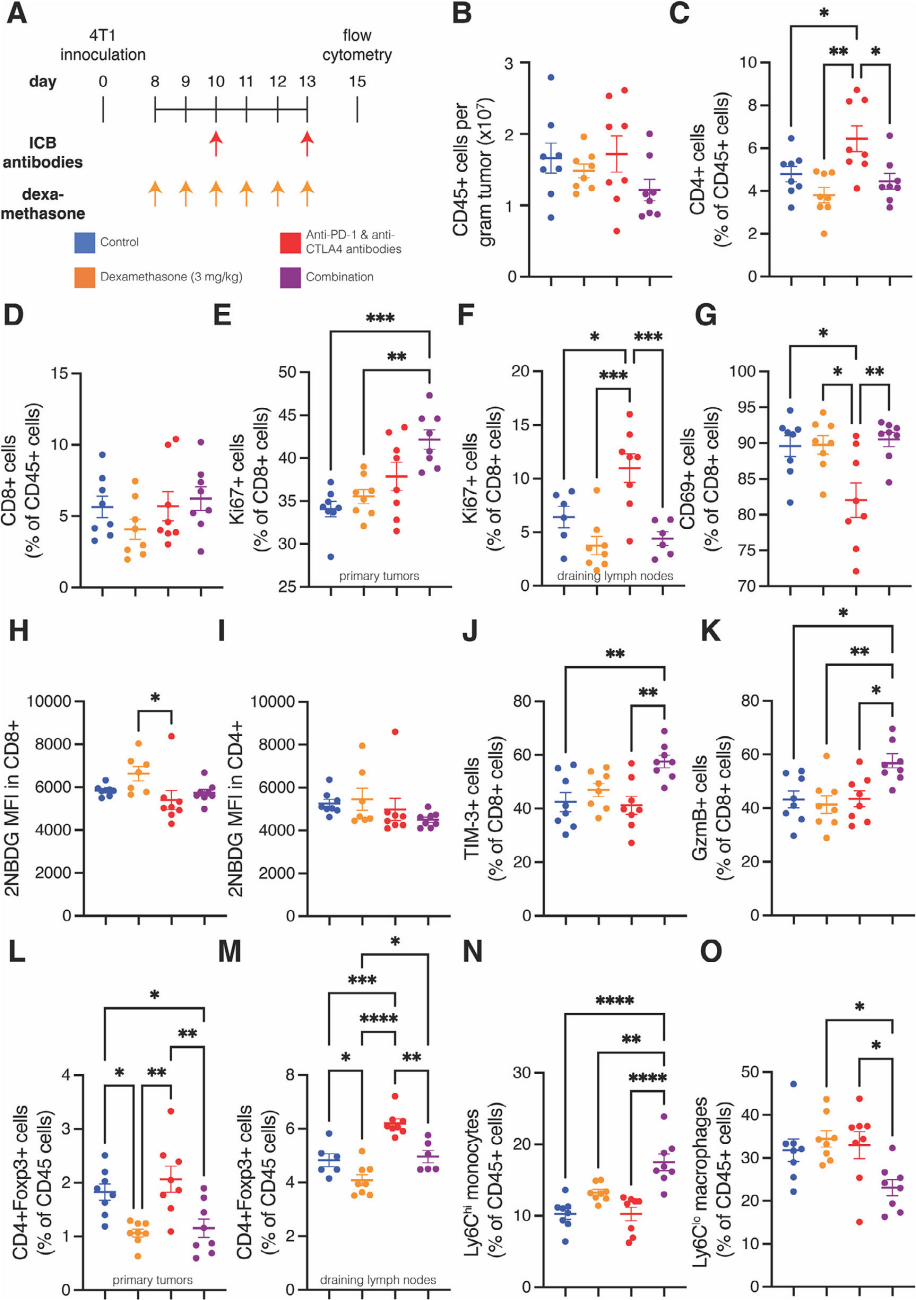
Dexamethasone enhances T cell activity and reduces immunosuppression in 4T1 tumors. A) Experiment scheme. Mice were inoculated with 4T1 cells in their mammary fat pad. On day 8, daily dexamethasone treatment was initiated, and on days 10 and 13 the immune checkpoint blockade (ICB) cocktail was administered. Tumors were collected on day 15 for flow cytometry. B) Quantification of CD45+ leukocytes per gram tumor. C) Quantification of the percentage of CD4+ T cells in the CD45+ leukocyte population of primary tumors. D) Quantification of the percentage of CD8+ T cells in the CD45+ leukocyte population of primary tumors. E) Quantification of the percentage of proliferating (Ki67+) cells in the CD8+ T cell population of primary tumors. F) Quantification of the percentage of Ki67+ cells in the CD8+ T cell population of draining lymph nodes. G) Quantification of the percentage of CD69+ cells in the CD8+ T cell population of primary tumors. H) Quantification of the mean florescence intensity (MFI) of the glucose analogue 2NBDG in CD8+ T cells of primary tumors. I) Quantification of the MFI of 2NBDG in CD4+ T cells of primary tumors. J) Quantification of the percentage of TIM‐3+ cells in the CD8+ T cell population of primary tumors. K) Quantification of the percentage of GzmB+ cells in the CD8+ T cell population of primary tumors. L) Quantification of the percentage of regulatory T cells (CD4+Foxp3+) in the CD45+ leukocyte population of primary tumors. M) Quantification of the percentage of CD4+Foxp3+ T cells in the CD45+ leukocyte population of draining lymph nodes. N) Quantification of the percentage of Ly6C^hi^ monocytes in the CD45+ leukocyte population of primary tumors. O) Quantification of the percentage of Ly6C^lo^ macrophages in the CD45+ leukocyte population of primary tumors. For all graphs, *n* = 8 mice per group and data plotted as average +/‐ standard error of the mean. Each dot represents one mouse. Statistical test by one‐way ANOVA with Holm‐Šídák's correction (^****^ denotes *P* < 0.0001, ^***^ denotes *P* < 0.001, ^**^ denotes *P* < 0.01, and ^*^ denotes *P* < 0.05).

We next investigated how these treatments affect the immune microenvironment by treating 4T1 tumor‐bearing mice with the same treatment groups and performing flow cytometry (**Figure** **2**A). We found that neither dexamethasone alone nor in combination with ICB affected the density of leukocytes in tumors (Figure 2B). However, dexamethasone as monotherapy or combination with ICB reduced the ICB‐induced expansion of the fraction of leukocytes that were CD4+ T cells (Figure 2C; Figure S3, Supporting Information). No treatment affected the fraction of CD8+ T cells (Figure 2D; Figure S4, Supporting Information). Interestingly, only the combination of dexamethasone and ICB increased the fraction of proliferating CD8+ intratumor T cells relative to controls (Figure 2E), while in the draining lymph node, the ICB group had a greater fraction of proliferating CD8+ T cells compared to each of the other groups (Figure 2F). These results suggest that dexamethasone enhances the trafficking of proliferating CD8+ T cells that were stimulated by ICB from the draining lymph node to the tumor.

We next investigated the differentiation of immune cells in 4T1 tumors. Dexamethasone did not affect the fractions of central memory and effector CD4+ T cells and CD8+ T cells (Figure S5; Supporting Information). However, while ICB alone reduced the fraction of CD69+ cells out of CD8+ T cells, adding dexamethasone to ICB returned the fraction to control levels (Figure 2G). These results indicate that dexamethasone does not reduce the earliest activation of T cells in the spleen (i.e., CD69 activation) and might enhance these cells’ infiltration into tumors. Dexamethasone did not affect the level of glucose analogue 2NBDG in CD8+ (Figure 2H) and CD4+ T cells (Figure 2I) indicating it does not affect glycolytic metabolism of these cells.

While T cells' expression of immune checkpoints occurs for other reasons besides an antigen encounter, we measured the expression to assess the frequency of T cells’ interactions with cancer cells. In primary tumors, the addition of dexamethasone to ICB increased the fraction of CD8+ T cells expressing the immune checkpoint TIM‐3 compared to control and ICB groups (Figure 2J). Additionally, the combination group demonstrated potentially enhanced activity of T cells, because these tumors had a higher percentage of granzyme B‐expressing T cells evaluated ex vivo compared to each other groups (Figure 2K). Thus, these data suggest that T cells in tumors treated with dexamethasone and ICB encountered cancer cells at a higher rate and had more potential for activity than in tumors treated with ICB monotherapy.

Dexamethasone also contributed to the depletion of immunosuppressive cells. Dexamethasone alone or in combination with ICB reduced the fraction of immunosuppressive regulatory T cells in the primary tumors (Figure 2L). Dexamethasone did the same in draining lymph nodes, as it reversed the expansion of these cells by ICB (Figure 2M). The anti‐CTLA4 antibody clone we used has been reported to induce regulatory T cells depletion.^[^ ^32^ ^]^ Among myeloid cells (Figure S6, Supporting Information), we did not observe any effects on neutrophils and dendritic cells by dexamethasone alone or by adding it to ICB treatment (Figure S7, Supporting Information). However, we observed that adding dexamethasone to ICB increased the fraction of CD45+ leukocytes that were immunostimulatory Ly6C^hi^ monocytes (Figure 2N) and reduced the fraction that was immunosuppressive Ly6C^lo^ macrophages (Figure 2O). Thus, these data suggest dexamethasone potentiates the ability of ICB to enhance trafficking, antigen encounter, and cytotoxic potential of antitumor T cells while depleting immunosuppressive regulatory T cells and myeloid cells in 4T1 tumors.

### 2.3 Dexamethasone Enhances Intratumor Spatial Distribution of T Cells

**Figure 3:**
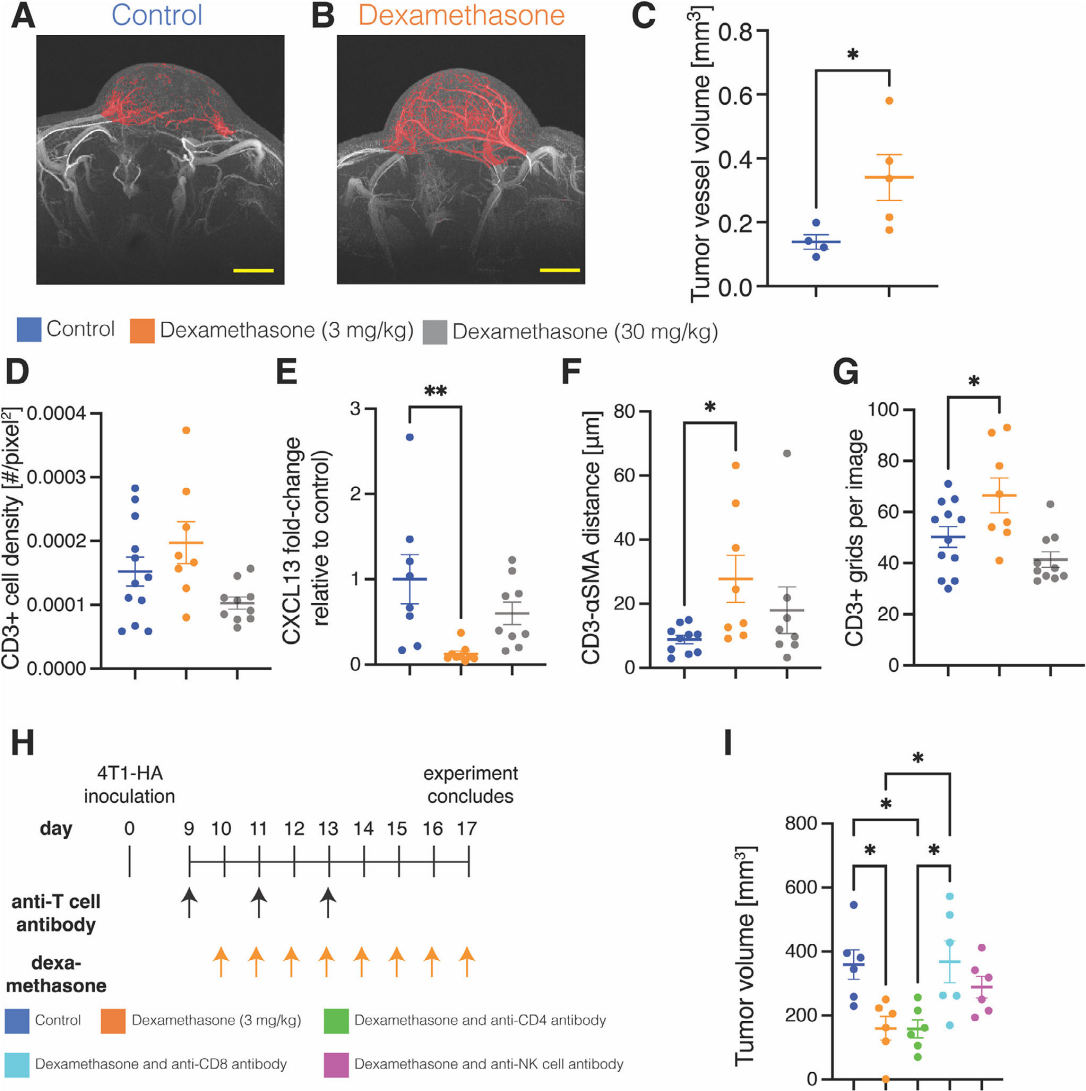
Dexamethasone ameliorates the heterogeneity of intratumor T cell spatial distribution. A,B) High resolution 3D magnetic resonance (MR) angiography images (maximum intensity projection mapping) of 4T1 breast tumors in (A) control‐treated and (B) daily 3 mg kg^−1^ dexamethasone‐treated tumors. Red color denotes tumor blood vessels as defined in the *MRI measurements* of the Method section. Due to spatial resolution limitations, this includes arterioles and venules larger than 50 µm but excludes capillaries. The yellow scale bar depicts 1 mm. C) Quantification of tumor vessel volume in MR angiography images. *n* = 4–5 mice. Statistical test by the unpaired *t*‐test. D) Quantification of CD3+ cell density assessed histologically. *n* = 3 mice, *N* = 8‐12 fields. Statistical test by one‐way ANOVA with Tukey's correction. E) Whole tumor mRNA expression of CXCL13 as measured by RT‐PCR. *n* = 8–9 mice per group. Statistical test by one‐way ANOVA with Tukey's correction. F) Quantification of CD3+ cell distance from αSMA assessed histologically. *n* = 3 mice per group, *N* = 8–12 fields. Statistical test by one‐way ANOVA with Tukey's correction. G) Quantification of the heterogeneity of spatial distribution of CD3+ T cells. The more CD3+ grids per image, the more homogeneous the T cell distribution. *N* = 3 mice per group, *N* = 8–12 fields. Statistical test by one‐way ANOVA with Tukey's correction. H) Experimental scheme for assessing the 4T1‐HA tumor growth delay with daily 3 mg kg^−1^ dexamethasone and depletion of T cells. I) Quantification of tumor volume at the endpoint of the experiment (day 17). *n* = 6 mice per group. Data plotted as average +/‐ standard error of the mean. For all panels except I, statistical test by one‐way ANOVA with Tukey's correction ^****^ denotes *P* < 0.0001, ^***^ denotes *P* < 0.001, ^**^ denotes *P* < 0.01, and ^*^ denotes *P* < 0.05.

Given that dexamethasone increased the fraction of CD8+ T cells that expressed immune checkpoints, which indicates that the T cells reached cancer cells, we hypothesized that dexamethasone enhances the distribution of T cells. To distribute throughout tumors, T cells flow through perfused intratumor blood vessels.^[^ ^33^ ^]^ We previously demonstrated that antiemesis dexamethasone increases the density of mature blood vessels in tumors,^[^ ^15^ ^]^ which are more likely to be perfused. To investigate perfusion throughout the whole tumor non‐invasively, we performed in vivo magnetic resonance imaging (MRI) angiography in mice bearing 4T1 tumors treated with dexamethasone (Figure S8A, Supporting Information), which did not affect body weight (Figure S8B, Supporting Information) nor tumor volume (Figure S8C, Supporting Information), using a blood pool contrast agent (i.e., Gadolisome, 100 nm diameter liposomes modified with gadolinium‐based contrast agents).^[^ ^34^ ^]^ We obtained 3D tumor microvascular angiographies at 50 µm isotropic spatial resolution in vivo in both control (**Figure** **3**A; Figure S8D, and Movie S1, Supporting Information) and dexamethasone‐treated mice (Figure 3B; Figure S8E, and Movie S2, Supporting Information). We found that dexamethasone increased the tumor vessel volume (arteriole and venule) by 145% (Figure 3C). Thus, infiltrating T cells should have more access to cancer cells deep in tumors because of the increased tumor blood volume.

We next compared how dexamethasone at its antiemesis dose, which increases perfusion and mature vessel density,^[^ ^15^ ^]^ and a 10‐fold larger dose, which reduces mature vessel density,^[^ ^15^ ^]^ affect the inhibition of T cell migration. We hypothesized that while the antiemesis dose would enhance T cell migration, the 10‐fold larger dose would not. We first confirmed with immunofluorescence histological analysis of 4T1 tumors (Figure S9, Supporting Information) that dexamethasone did not reduce the density of CD3+ T cells (Figure 3D). Next, we assessed the mRNA levels of CXCL13. We previously reported dexamethasone reduces intratumor hyaluronan synthase mRNA expression levels, hyaluronan protein levels and hypoxia,^[^ ^15^ ^]^ Hyaluronan^[^ ^35^ ^]^ and hypoxia^[^ ^36^ ^]^ contribute to the production of CXCL13 in CAFs, which repulses lymphocytes.^[^ ^26^, ^36^, ^37^, ^38^ ^]^ We found whole‐tumor CXCL13 levels reduced with antiemesis dexamethasone treatment (Figure 3E). CXCL13 is expressed not only by CAFs but also by various immune cells. CXCL13 can also have an immune‐activating role, including in the formation of tertiary lymphoid structures.^[^ ^39^ ^]^ Then, we quantified the distance of CD3+ cells from their nearest αSMA+ cell, which includes perivascular cells and myofibroblasts, and found that T cells migrated widely. Specifically, antiemesis dexamethasone increased the average distance between each CD3+ cell and its nearest αSMA+ cell (Figure 3F). We binned the distances to the nearest αSMA+ area for each CD3+ cell and found that antiemesis dexamethasone increased the proportion of CD3+ cells >20 µm from the nearest αSMA+ area (Figure S10, Supporting Information).

Then, we measured heterogeneity in T cell spatial distribution throughout the tumor parenchyma. To test this, we divided the immunofluorescence images into 100 pixel‐sided square grids^[^ ^28^, ^40^ ^]^ and counted how many grids had CD3+ T cells present. We found that images from antiemesis dexamethasone treated tumors had CD3+ T cells in the most grids thereby indicating a wider and more homogenous distribution (Figure 3G). We performed similar analyses of tumor‐associated macrophages in these 4T1 tumors and found no difference in densities nor in distribution (Figure S11, Supporting Information). Thus, dexamethasone increases the transport of T cells by blood vessels and through CAFs leading to increased homogeneity of T cell distribution and encounter of intratumor antigens.

We reasoned that if antitumor T cells transited to cancer cells more effectively with dexamethasone treatment, as our data suggests, then dexamethasone monotherapy should exert an antitumor effect in tumor models with effectively trained cytotoxic T lymphocytes. Thus, we tested the efficacy of dexamethasone with a model cancer cell line that constitutively expresses a foreign antigen. In such a model, starting from cancer cell inoculation, the host immune system would encounter a high‐affinity antigen expressed by all cancer cells to which T cells could bind. As a result, antitumor T cells would be primed before the initiation of dexamethasone treatment, and they would only need to overcome transiting and immunosuppressive barriers in the TME to exert an antitumor effect. We inoculated mice with the breast cancer cell line 4T1‐HA, which expresses influenza hemagglutinin (HA) as a model high‐affinity tumor antigen and treated with dexamethasone either with or without depleting antibodies against CD4+ T cells, CD8+ T cells, and NK cells (Figure 3H). Although 4T1 tumor growth is unaffected by dexamethasone,^[^ ^15^ ^]^ we found dexamethasone monotherapy induced 4T1‐HA tumor growth delay (Figure 3I). This growth delay was abrogated by CD8+ T cell depletion but not CD4 T+ cell depletion. Dexamethasone is not toxic to 4T1‐HA cells (Figure S12, Supporting Information) nor to 4T1 cells^[^ ^15^ ^]^ in vitro thereby supporting the notion that the antitumor activity is related to the immune response rather than direct effects on cancer cells. Overall, these data are consistent with the hypothesis that dexamethasone enables cytotoxic T lymphocytes to more effectively reach and kill cancer cells.

### 2.4 Dexamethasone Potentiates ICB‐Induced Infiltration of Antigen‐Specific T Cells

**Figure 4:**
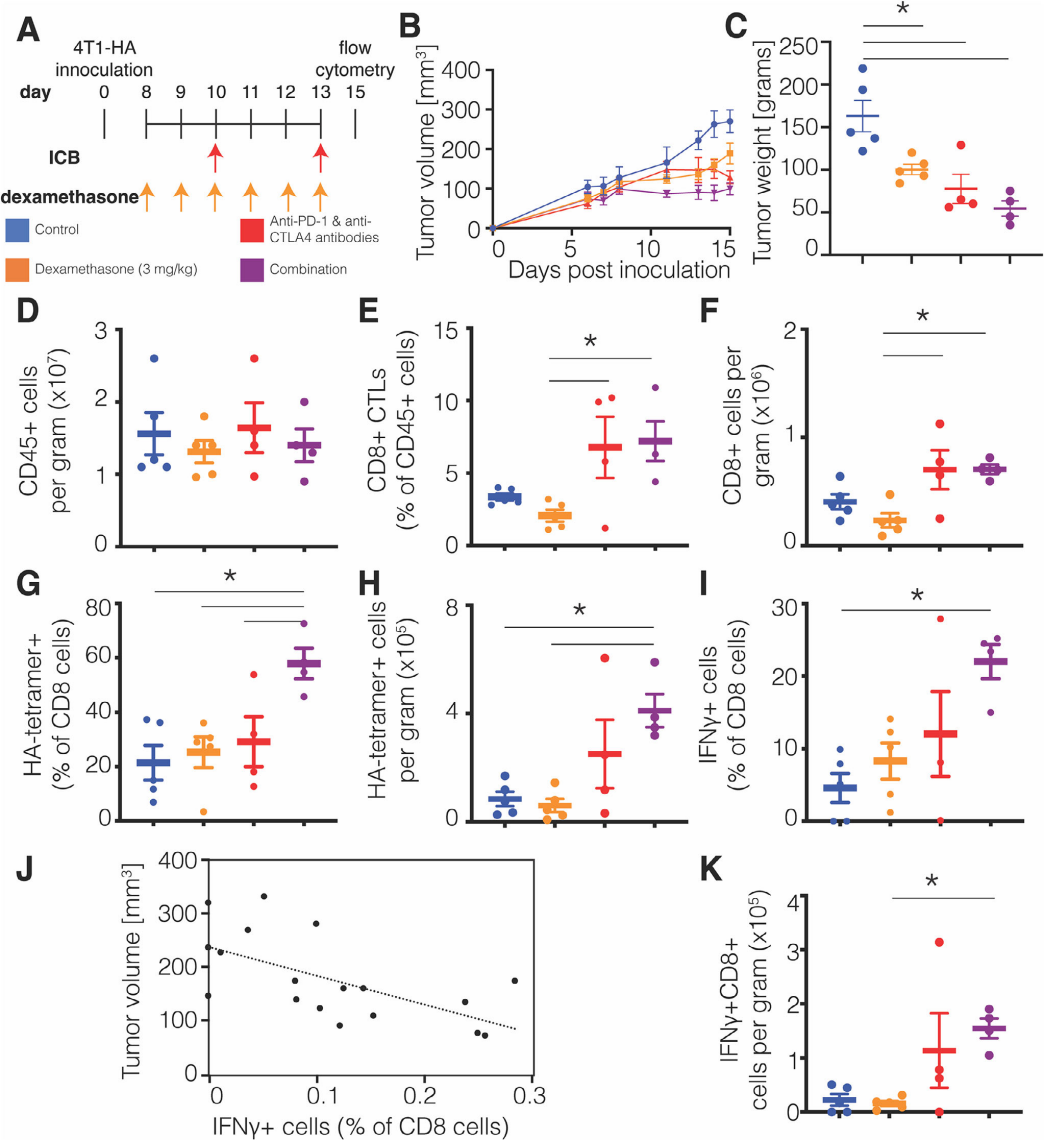
Dexamethasone potentiates ICB‐induced enrichment and activity of antigen‐specific T cells in tumors. A) Experiment scheme. Mice were inoculated with 4T1‐HA cells in their mammary fat pad. On day 8, daily dexamethasone treatment was initiated and on days 10 and 13 the immune checkpoint blockade (ICB) cocktail was administered. Tumors were collected on day 15 for flow cytometry. B) Graph of tumor volume over time throughout the experiment. C) Quantification of tumor volume on the day of treatment completion. D) Quantification of CD45+ leukocytes per gram tumor. E) Quantification of the percentage of CD8+ cytotoxic T lymphocytes (CTLs) in the CD45+ leukocyte population. (F) Quantification of CD8+ T cells per gram tumor. G) Percentage of HA‐tetramer+ cells in the CD8+ T cell population. H) HA‐tetramer+CD8+ T cells per gram tumor. I) Percentage of IFNγ+ cells in the CD8+ T cell population. J) Correlation of tumor volume on day 15 and percentage of IFNγ+ cells in the CD8 T cell population fit to a linear equation (R^2^ = 0.39). K) Number of IFNγ+CD8+ T cells per gram tumor. For all studies, *n* = 4−5 mice per group. Data plotted as average +/‐ standard error of the mean. Each dot represents one mouse. Statistical test by one‐way ANOVA with Holm‐Šídák's correction (^*^ denotes *P <* 0.05).

We further investigated how dexamethasone alone and combined with ICB affected antigen‐recognizing T cells using flow cytometry. This time we combined dexamethasone with two cycles of an ICB cocktail (**Figure** **4**A). We monitored 4T1‐HA tumor volume over time (Figure 4B; Figure S13, Supporting Information). Tumors in dexamethasone monotherapy, ICB cocktail and the combination groups all had less weight on day 15 compared to controls (Figure 4C).

As in the 4T1 tumor model, we found no change in the density of CD45+ tumour‐infiltrating immune cells in 4T1‐HA tumors (Figure 4D). Although ICB increased CD8+ T cell infiltration relative to dexamethasone monotherapy, the addition of dexamethasone to ICB had no effect (Figure 4E,F; Figure S14, Supporting Information). We measured the infiltration of tumor antigen‐specific CD8+ T cells in these mice using H‐2K^d^ Influenza HA tetramer (HA‐tetramer) and found the percentage of HA‐tetramer+ cells among CD8+ T cells was increased in the combination group compared to each other group (Figure 4G; Figure S15, Supporting Information). The density of HA‐tetramer+CD8+ T cells was increased only in the tumors of combination‐treated mice relative to controls and dexamethasone monotherapy (Figure 4H). These results indicate that dexamethasone increased tumor antigen‐specific CD8+ T cell infiltration, which ICB monotherapy could not induce alone.

We also examined the induction of HA‐specific CD8+ T cell response by intracellular IFNγ staining after stimulation and found that the percentage of HA‐specific IFNγ‐producing cells was increased compared to controls only by the combination therapy of dexamethasone and ICB (Figure 4I; Figure S16, Supporting Information). This measurement correlated with tumor volume on day 15 (Figure 4J), which indicates these cells play a role in the antitumor response in this model. Similarly, the density of HA‐specific IFNγ‐producing cells was increased only by the combination (Figure 4K). The weights of the spleens of the dexamethasone‐treated groups were reduced, reflecting both a toxic effect on lymphoid organs and suppression of tumor growth (Figure S17, Supporting Information). Thus, dexamethasone potentiates the ICB‐induced infiltration and activity of antigen‐specific T cells.

### 2.5 Dexamethasone Suppresses – Not Eliminates – Vaccine Stimulation of Tumor‐Specific T Cells’ Proliferation, Differentiation, and Activity in Antigen‐Naïve Hosts

**Figure 5:**
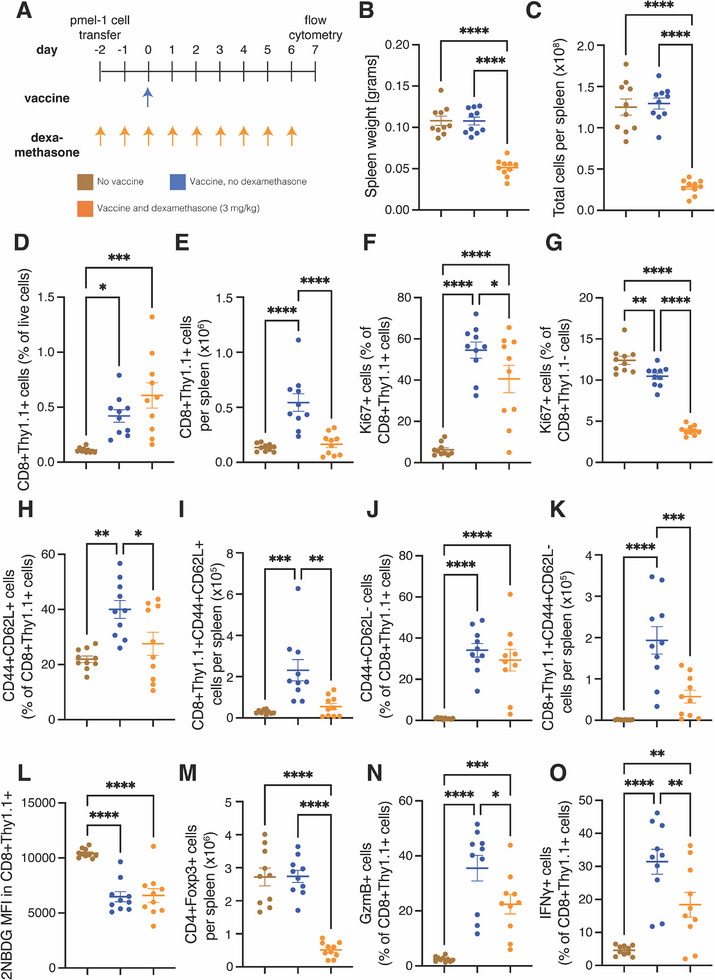
Dexamethasone does not completely suppress tumor‐specific T cell activities in antigen‐naïve mice. A) Experimental scheme. Healthy mice were administered activated pmel‐1 T cells labeled with the congenic marker Thy1.1 on day ‐2. From that day until day 6, the mice were administered 3 mg/kg dexamethasone daily. On day 0, they were administered a gp100 vaccine. Spleens were harvested for flow cytometry on day 7. B) Weight of spleens on day 7. C) Total number of cells per spleen. (D) Tumor‐specific (CD8+Thy1.1+) T cells as a percentage of live cells. E) The number of CD8+Thy1.1+ T cells per spleen. F) The fraction of proliferating (Ki67+) CD8+Thy1.1+ T cells. G) The fraction of Ki67+ host (CD8+Thy1.1‐) T cells. H) The fraction of central memory (CD44+CD62L+) CD8+Thy1.1+ T cells. I) The number of CD44+CD62L+CD8+Thy1.1+ T cells per spleen. J) The fraction of effector memory (CD44+CD62L‐) CD8+Thy1.1+ T cells. K) The number of CD44+CD62L‐CD8+Thy1.1+ T cells per spleen. L) The mean florescence intensity (MFI) of the glucose analogue 2NBDG in CD8+Thy1.1+ T cells. (M) The number of regulatory T cells (CD4+Foxp3+) per spleen. N) The fraction of granzyme B (GzmB)‐expressing CD8+Thy1.1+ ex vivo T cells. O) The fraction of interferon gamma (IFNγ)‐expressing CD8+Thy1.1+ T cells after stimulation. For all graphs, *n* = 10 mice per group and data plotted as average +/‐ standard error of the mean. Each dot represents one mouse. Statistical test by one‐way ANOVA with Holm‐Šídák's correction (^****^ denotes *P* < 0.0001, ^***^ denotes *P* < 0.001, ^**^ denotes *P* < 0.01, and ^*^ denotes *P* < 0.05).

After observing that dexamethasone spared antigen‐specific immune cells while depleting immunosuppressive T cells in tumors and their draining lymph nodes, we hypothesized that dexamethasone differently affects immune cells of various antigen experience throughout the host. We chose to first investigate how dexamethasone affects cells in lymphoid organs during vaccine stimulation in antigen‐naïve, non‐tumor bearing hosts. In this model system, we aimed to observe how dexamethasone would affect T cells that are encountering tumor antigens for the first time. Introducing tumor cells into the study would introduce antigens into the host and make the results harder to interpret. Specifically, we tested whether dexamethasone would diminish the antigen‐specific expansion of adoptively transferred naïve pmel‐1 T cells, which express the T cell receptor against melanocyte differentiation antigen gp100 overexpressed in human melanoma, in response to cognate antigen stimulation. We adoptively transferred naïve pmel‐1 T cells expressing the congenic marker Thy1.1 on day ‐2, administered dexamethasone daily between days ‐2 and 6, and injected a hgp100 peptide pulsed dendritic cell vaccine^[^ ^41^ ^]^ on Day 0 before collecting the spleens on Day 7 (**Figure** **5**A) for flow cytometry (Figure S18, Supporting Information). We first confirmed that dexamethasone reduced the spleen weight (Figure 5B) and number of cells in the spleen (Figure 5C), as shown in other reports.^[^ ^42^ ^]^ While dexamethasone did not affect the fraction of live cells in the spleen that are antigen stimulated, i.e., tumor‐specific Thy1.1+CD8+ cells, induced by vaccine administration (Figure 5D), it reduced the total amount (Figure 5E). Still, adding dexamethasone to the vaccine resulted in a 5.4‐fold greater fraction of proliferating antigen‐stimulated Thy1.1+CD8+ cells in spleens than in non‐vaccinated controls (Figure 5F). In contrast, adding dexamethasone to the vaccine reduced the fraction of proliferating unstimulated T cells (i.e., Thy1.1‐CD8+ cells) in the spleen by more than one‐third compared to controls (Figure 5G). Thus, in mice naïve to the tumor antigen at time of dexamethasone initiation, tumor‐specific T cells respond to vaccination and expand with reduced capacity while non‐stimulated T cells’ proliferation is stifled.

We next tested whether dexamethasone affected differentiation and activation into memory T cells. We found that dexamethasone reduced to control levels the fraction (Figure 5H) and total number of CD44+CD62L+ central memory cells (Figure 5I) induced by the vaccine. In contrast, dexamethasone did not reduce the fraction (Figure 5J) but did reduce the total number of CD44+CD62L‐ effector memory cells (Figure 5K). We found that dexamethasone affected CD69+ resident tumor‐specific T cells similarly to CD44+CD62L‐ effector memory cells (Figure S19, Supporting Information). T cell differentiation shifts glucose metabolism, with naïve T cells but not differentiating memory T cells requiring it. We found that adding a daily dexamethasone treatment to the vaccine did not affect the level of glucose analogue 2NBDG, thereby demonstrating metabolically that dexamethasone did not hamper differentiation (Figure 5L). Additionally, dexamethasone treatment reduced the fraction (Figure S20, Supporting Information) and total number of regulatory T cells compared to both control and vaccinated groups (Figure 5M). Thus, dexamethasone before vaccination suppressed, without eliminating tumor‐specific T cell differentiation while depleting the spleens of regulatory T cells.

We next tested whether dexamethasone administered in antigen‐naïve mice affects markers of tumor‐specific T cell function. In an ex vivo analysis, we found that the addition of dexamethasone to the vaccine reduced by less than half the fraction of Granzyme B+ cells amongst tumor‐specific T cells, and the reduced fraction was still an order of magnitude higher than cells from unvaccinated mice (Figure 5N). We observed similar results in the fraction of IFNγ‐expressing T cells amongst tumor‐specific T cells after stimulation in vitro (Figure 5O). There is almost no checkpoint expression in the tumor‐specific T cells, underscoring that this model is an acute model rather than the chronic case of T cell exhaustion in patients (Figure S21, Supporting Information). Thus, under daily dexamethasone treatment initiated before vaccination, the fractions and amounts of regulatory T cells and proliferating host CD8+ T cells were reduced compared to unvaccinated mice. In contrast, the proliferation, differentiation, activation into memory, and function of tumor‐specific CD8+ T cells was enhanced in vaccinated mice with dexamethasone compared to unvaccinated mice. Nonetheless, these processes occurred at a suppressed rate compared to that in vaccinated mice not receiving dexamethasone.

### 2.6 Dexamethasone Suppresses Vaccine Stimulation of Tumor‐Specific T Cells’ Proliferation and Differentiation but not Activity in Antigen‐Experienced Hosts

**Figure 6:**
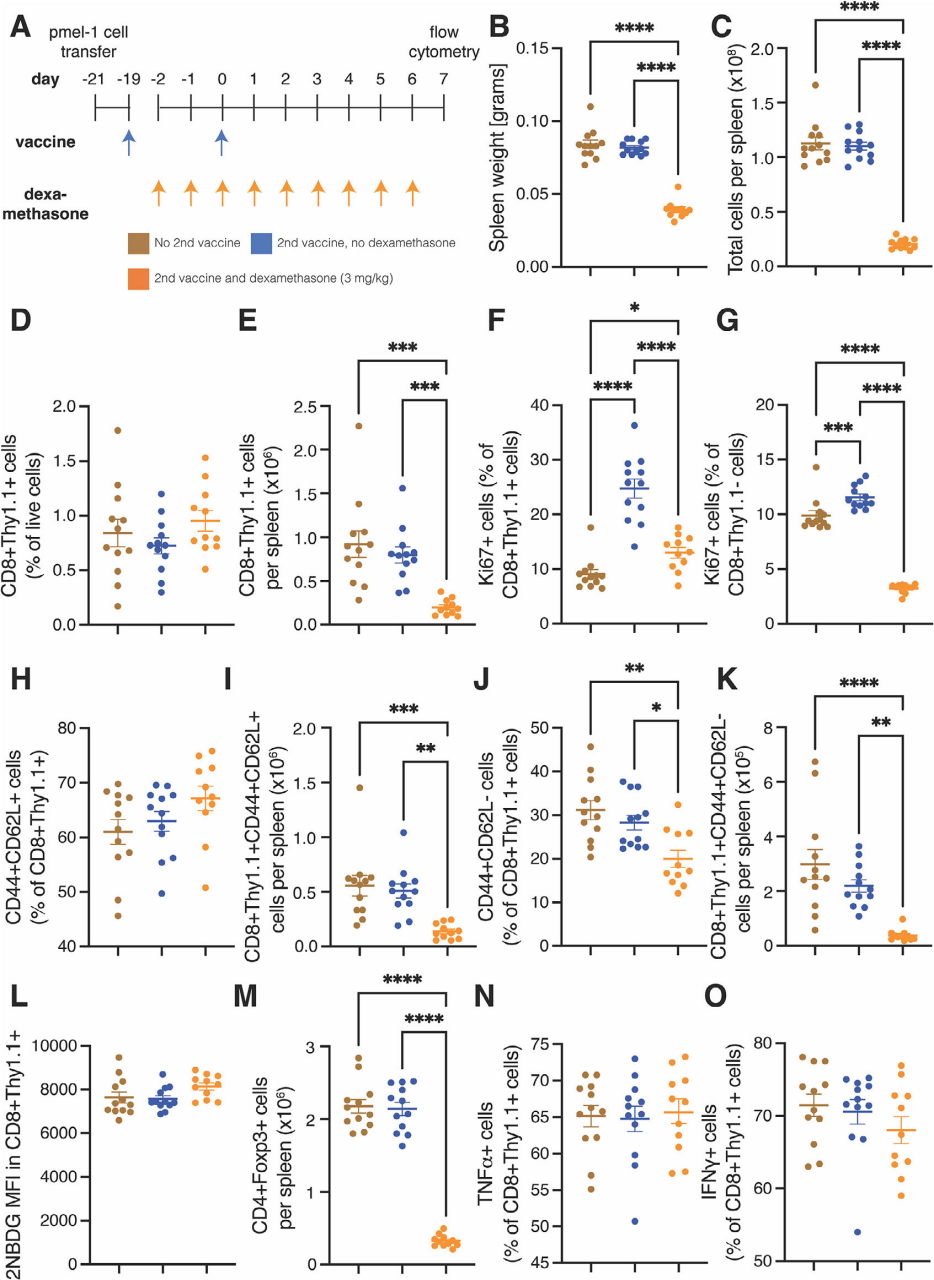
Dexamethasone suppresses tumor‐specific T cell proliferation and differentiation but not activity in antigen‐experienced mice. A) Experimental scheme. Healthy mice were administered activated pmel‐1 T cells labeled with the congenic marker Thy1.1 on day ‐21 and vaccinated for the first time with a gp100 vaccine on day ‐19. From day ‐2 until day 6, the mice were administered 3 mg/kg dexamethasone daily. On day 0, they were administered the vaccine a second time. Spleens were harvested for flow cytometry on day 7. B) Weight of spleens on day 7. C) Total number of cells per spleen. D) Tumor‐specific (CD8+Thy1.1+) T cells as a percentage of live cells. E) The number of CD8+Thy1.1+ T cells per spleen. F) The fraction of proliferating (Ki67+) CD8+Thy1.1+ T cells. G) The fraction of Ki67+ host (CD8+Thy1.1‐) T cells. H) The fraction of central memory (CD44+CD62L+) CD8+Thy1.1+ T cells. I) The number of CD44+CD62L+CD8+Thy1.1+ T cells per spleen. (J) The fraction of effector memory (CD44+CD62L‐) CD8+Thy1.1+ T cells. K) The number of CD44+CD62L‐CD8+Thy1.1+ T cells per spleen. L) The mean florescence intensity (MFI) of the glucose analogue 2NBDG in CD8+Thy1.1+ T cells. M) The number of regulatory T cells (CD4+Foxp3+) per spleen. N) The fraction of tumor necrosis factor α (TNFα)‐expressing CD8+Thy1.1+ T cells after stimulation. O) The fraction of interferon gamma (IFNγ)‐expressing CD8+Thy1.1‐ T cells after stimulation. For all graphs, *n* = 11–12 mice per group and data plotted as average +/‐ standard error of the mean. Each dot represents one mouse. Statistical test by one‐way ANOVA with Holm‐Šídák's correction (^****^ denotes *P* < 0.0001, ^***^ denotes *P* < 0.001, ^**^ denotes *P* < 0.01, and ^*^ denotes *P* < 0.05).

We next investigated the effect of dexamethasone during vaccine stimulation in antigen‐experienced mice. In this model system, we aimed to observe how dexamethasone affects T cells that have already encountered tumor antigens. To test this, we adoptively transferred naïve pmel‐1 T cells expressing the congenic marker Thy1.1 followed by a hgp100 peptide pulsed dendritic cell vaccine two days later. After three weeks, we administered dexamethasone daily between days ‐2 and 6, injected this experiment's second hgp100 peptide pulsed dendritic cell vaccine (37) on Day 0, and collected the spleens on Day 7 (**Figure** **6**A). As in the antigen‐naïve experiment, we found that the addition of dexamethasone to the protocol reduced the weight of the spleens (Figure 6B) and the total live cells in the spleen (Figure 6C). The second vaccination did not affect spleen weight nor cell amount. Neither the second vaccination alone nor combined with dexamethasone affected the fraction of tumor antigen‐specific cells (Figure 6D), but the addition of dexamethasone reduced the total number of these cells (Figure 6E). As in the antigen‐naïve experiment, the addition of dexamethasone only partly abrogated the increased fraction of antigen‐stimulated T cells proliferating that the second vaccination induced (Figure 6F), and it reduced the fraction of unstimulated T cells proliferating compared to both the control group without a second vaccination and the one with a second vaccination (Figure 6G). These findings indicate that, under dexamethasone treatment in antigen‐experienced mice, tumor‐specific T cells’ proliferation is maintained compared to that of unstimulated T cells.

We next tested whether adding dexamethasone to the second vaccination affected differentiation and activation into memory T cells in antigen‐experienced mice. We found that dexamethasone did not affect the fraction (Figure 6H) but did reduce the total number of CD44+CD62L+ central memory cells induced compared to the once‐ or twice‐vaccinated groups (Figure 6I). In contrast, the addition of dexamethasone reduced both the fraction (Figure 6J) and the total number of CD44+CD62L‐ effector memory cells (Figure 6K). We found that dexamethasone affected CD69+ resident tumor‐specific T cells similarly to CD44+CD62L+ tumor‐specific cells (Figure S22, Supporting Information). Given that in this antigen‐experienced model there were fewer naïve T cells, which require glucose metabolism, there were lower levels of 2NBDG (Figure 6L). A second vaccination did not affect the metabolism, and as in the experiment of antigen‐naïve mice, the addition of dexamethasone did not affect the metabolism, either (Figure 6L), suggesting that dexamethasone did not affect differentiation. As in the previous experiment, daily dexamethasone treatment reduced the fraction (Figure S23, Supporting Information) and total number of regulatory T cells (Figure 6M). Thus, under dexamethasone treatment in an antigen‐experienced model, immunosuppressive regulatory T cells are depleted while differentiation and activation into memory T cells occurs but is suppressed.

Unlike in the antigen‐naïve experimental model, dexamethasone administration did not affect markers of tumor‐specific T cell function. Under in vitro stimulation, the addition of daily dexamethasone did not affect the fraction of TNFα‐ (Figure 6N) and IFNγ‐expressing cells in the antigen‐stimulated T cell population (Figure 6O). Like the antigen‐naïve model, tumor‐specific T cells did not express checkpoints (Figure S24, Supporting Information), indicating this is an acute rather than a chronic model. Thus, in this antigen‐experienced model, the addition of dexamethasone suppressed – but did not eliminate – tumor‐specific T cell proliferation and differentiation. Specifically, adding dexamethasone reduced the amounts of cells in spleens, but there was more depletion of host CD8+ T cells and regulatory T cells compared to the restriction of expansion of tumor‐specific T cells by vaccination. Dexamethasone did not affect markers of tumor‐specific T cell activity in the spleens of antigen‐experienced mice.

## 3 Discussion

Our results highlight the potential benefits of using antiemetic dexamethasone in combination with ICB in murine models of primary breast tumors and their spontaneous metastases. The mechanism is multifactorial. First, immunosuppressive signaling in the TME is reduced. Second, immunosuppressive cells are depleted systemically without eliminating antigen‐experienced cells. Third, the movement of antitumor cells is promoted from the draining lymph node to and throughout the tumor. These factors are connected, as lymphocytes throughout the host (e.g., in the spleen and thymus) can be better trained on tumor antigens. Once lymphocytes reach a cancer cell, their anticancer activities are likely to be less inhibited by resident immunosuppressive cells and their signaling in the TME. We expect that the benefits of dexamethasone to ICB are sensitive to dose, length of treatment course and tumor type, as more intense regimens of steroids exacerbate TME immunosuppression. Indeed, longer treatment with a higher dose of steroids blocked efficacy of ICB in brain tumors.^[^ ^4^ ^]^

Dexamethasone had varied effects on immune cells, which interact to alter antitumor immunity induced by ICB. Protumor regulatory T cells in 4T1 primary tumors and their draining lymph nodes were depleted despite stimulation from ICB. Systemically, while host and regulatory T cells were depleted more than tumor‐specific T cells, markers of activity in tumor‐specific T cells were unaffected. Higher doses and longer regimens might deplete host T cells too extensively.^[^ ^43^ ^]^ Additionally, the fraction of CD45+ leukocytes were shifted from immunosuppressive Ly6C^lo^ macrophages to immunostimulatory Ly6C^hi^ monocytes.^[^ ^43^, ^44^ ^]^ The addition of dexamethasone to the ICB cocktail enriched the fraction of CD8+ T cells expressing immune checkpoints, which indicates exhaustion and that the cell was in contact with tumor antigens. CD8+ T cells expressing granzyme B were also enriched, which suggests a higher cytotoxic potential. By enriching tumors of anticancer T cells, and depleting tumor‐supporting immune cells that would T cells’ activities, dexamethasone promoted the efficacy of ICB treatment.

Infiltrating immune cells must traffic to and within tumors through perfused blood vessels and migrate to cancer cells.^[^ ^33^ ^]^ There are several ways dexamethasone affects T cell migration in tumors. First, we previously found that the antiemesis regimen of dexamethasone presented in the current work reduces VEGF, ANG2, and VEGFR2 expression.^[^ ^15^ ^]^ Reducing angiogenic factors would increase the transmigration of circulating lymphocytes into tumors.^[^ ^44^, ^45^ ^]^ Second, we previously found that this antiemesis regimen of dexamethasone reduced tumor tissue stiffness.^[^ ^15^ ^]^ Reducing stiffness increases T cell migration^[^ ^46^ ^]^ and CD8+ T cell activity.^[^ ^47^ ^]^ Third, we previously found that this antiemesis regimen of dexamethasone reduces hypoxia. Reduced hypoxia correlates with increased T cell migration.^[^ ^48^, ^49^ ^]^ High doses of dexamethasone prune tumor vessels,^[^ ^15^ ^]^ likely adversely affecting T cell migration. Accordingly, reduced stiffness^[^ ^50^ ^]^ and increased perfusion^[^ ^23^, ^30^, ^45^ ^]^ are potential predictive biomarkers of response to ICB.

The increase in volume of perfused blood vessels is associated with reduced hypoxia and immunosuppressive signaling in the primary tumor. The lack of vascular normalization at lower and higher doses of dexamethasone^[^ ^15^ ^]^ suggests that this effect is dose‐sensitive. As a result of increased perfusion, fewer immunosuppressive cytokines reach the tumor‐draining lymph node and antigen‐presenting cells can more readily capture tumor antigens and migrate to the lymph node. In turn, antigen‐recognizing cytotoxic lymphocytes can more readily activated and primed in the tumor‐draining lymph node. Then, because of increased volume of perfused blood vessels, these antigen‐recognizing cytotoxic lymphocytes can more readily transit back to the primary tumor and migrate to the tumor parenchyma. Finally, these antigen‐recognizing cytotoxic lymphocytes can more readily proliferate, because the tumor is less immunosuppressive.

There are limitations and factors to consider. Even though we tested the efficacy of the combination therapy in spontaneous models of metastasis that can take more than a month to form, murine cancer models are acute thereby reducing the effects of T cell exhaustion. Our study did not explore how dexamethasone affects either anti‐PD‐1 or anti‐CTLA‐4 monotherapies. The treatment protocol in the current study only lasted for one week, but some patients take dexamethasone for longer periods, such as those with tuberculosis and autoimmune diseases. Future studies should investigate the systemic effects of long‐term dexamethasone, the toxicities when combined with ICB, and whether normalization can be sustained. Understanding how dexamethasone affects cell‐specific secretion of inflammatory factors, such as CXCL13, is essential. Even though our study included testing of two primary breast tumor models (4T1, E0771) and their spontaneous metastases, much of the mechanistic studies were performed with one breast cancer cell line. Clinical data demonstrating the negative effects of dexamethasone on ICB efficacy is in lung cancer, which is more sensitive to ICB monotherapy.^[^ ^3^, ^11^, ^12^ ^]^ Future studies should investigate the tumor type specific effects of dexamethasone.

Our results provide a rationale for continued use of dexamethasone for highly emetogenic chemotherapy in combination with ICB. A future challenge involves investigating whether antiemetic protocols of dexamethasone could be modified to improve the efficacy of ICB. This involves balancing the dose‐ and scheduling‐dependencies of dexamethasone's various effects: antiemesis, systemic immunosuppression and tumor immune cell infiltration. Retrospective studies could compare outcomes with different protocols, while prospective studies could test immune cell infiltration before and treatment by biopsy. Unbiased mechanistic profiling should be undertaken to provide a more comprehensive framework regarding how dexamethasone affects intratumor immune cells. A spatial transcriptomics/proteomics approach could corroborate the homogenous distribution of lymphocytes we observed and elucidate further details about the underlying mechanisms. Imaging of perfusion longitudinally could be a potential biomarker of response to dexamethasone.^[^ ^23^, ^30^, ^50^ ^]^

## 4 Experimental Section

### Animal Tumor Models

Female, 6‐week‐old BALB/c (for 4T1 studies) and C57BL/6 (for E0771 studies) female mice were purchased from Charles River Co. (Tokyo, Japan). All the experiments were conducted under the ethical guidelines of The University of Tokyo, of the Innovation Center of NanoMedicine (Kawasaki, Japan), and of the Republic of Cyprus and the European Union under a license acquired by the Cyprus Veterinary Services (No CY/EXP/PR.L1/2014), the Cyprus national authority for monitoring animal research. The cells used in the studies were 4T1 (ATCC CRL‐2539) and E0771 (94A001, CH3 BioSystems) from ATCC and CH3 BioSystems, respectively. The cells were maintained at 37 °C/ 5% CO_2_ in RPMI‐1640 (LM‐R1637, biosera) supplemented with 10% fetal bovine serum (FBS, FB‐1001H, biosera) and 1% antibiotics (A5955, Sigma). The cells were confirmed to be mycoplasma free. The 4T1 cells expressing the influenza HA gene were made by retroviral transfection with MSCV‐IRES‐GFP vector containing HA cDNA from the Mount Sinai strain of the PR8 influenza virus. The expression of HA protein on the cell surface was confirmed by flow cytometry using HA‐specific mAb, H18, and PE‐ or eFluor 660‐conjugated F(ab')_2_ fragment of goat anti‐mouse IgG polyclonal antibody (eBioscience, San Diego, CA). Orthotopic models for murine mammary tumors were generated by implantation of 5 × 10^4^ (4T1), 1 × 10^6^ (4T1‐HA) or 1 × 10^5^ (E0771) mouse mammary cancer cells in 40 µL of serum‐free medium into the third mammary fat pad of the mice. For the 4T1 studies, once tumors grew past 6 mm in diameter, they were extracted, cut into 1 mm^3^ chunks and implanted for all in vivo studies. Serial solid passages were limited to five times.

### Pmel‐1 Adoptive Cell Transfer Flow Cytometry

Pmel‐1‐TCR transgenic mice recognizing the H‐2D^b^‐restricted human gp100 peptide (hgp100 25–33, KVPRNQDWL) were obtained from The Jackson Laboratory (Bar Harbor, ME). The strain is also homozygous for the Thy1a (Thy1.1) allele. For treatment of antigen‐naïve mice with dexamethasone, C57BL/6 mice were intravenously injected with 1 × 10^7^ naïve spleen cells from pmel‐1 TCR Tg mice on day ‐2. Dexamethasone (0.06 mg in 100 µL PBS, equivalent to 3 mg kg^−1^ in a mouse with 20 g body weight) was administered by intraperitoneal (i.p.) injection from day ‐2 to day 6 daily. A hgp100 peptide‐pulsed bone marrow‐derived dendritic cell (DC) vaccine was prepared as before.^[^ ^37^ ^]^ Specifically, bone marrow cells from femurs and tibias were cultured in RPMI‐1640 supplemented with 10% FBS, 12.5 mm HEPES, 5 × 10^−5^ m 2‐mercaptoethanol, 1 × 10^−5^ m sodium pyruvate, 1% nonessential amino acids, 100 U mL^−1^ penicillin, 100 µg mL^−1^ streptomycin and 20 ng mL^−1^ GM‐CSF (PeproTech, Rocky Hill, NJ) for 8 days. DCs were stimulated with 1 µg mL^−1^ lipopolysaccharide (Wako Pure Chemical) for 16 h and pulsed with hgp100 peptide at 1 µg mL^−1^ for 2 h. Then, DCs (1 × 10^6^) were subcutaneously injected into the flank of mice to immunize mice on day 0. On day 7 after DC vaccination, spleens were harvested. For treatment of antigen‐experienced mice with dexamethasone, C57BL/6 mice were administered naive pmel‐1 T cells labeled with the congenic marker Thy1.1 on day ‐21 and vaccinated for the first time with a gp100 vaccine on day ‐19 and day 0. From day ‐2 until day 6, the mice were administered 3 mg/kg dexamethasone daily. Spleens were harvested for flow cytometry on day 7.

Splenocytes were stained with Zombie Yellow (BioLegend, San Diego, CA) to eliminate dead cells. The cells were then pretreated with Fc Block (anti‐CD16/32 clone 2.4G2; BioXcell), stained with following mAbs: Pacific Blue‐conjugated anti‐CD3, APC/Cyanine7‐conjugated anti‐CD8, APC‐conjugated anti‐Thy1.1, FITC‐conjugated anti‐CD62L, PE/Cyanine7‐conjugated anti‐CD44, PE/Dazzle594‐conjugated anti‐B220, anti‐F4/80, anti‐NK1.1 and anti‐Ly6G, (BioLegend). Stained cell data were acquired on a Gallios flow cytometer (Beckman Coulter, Brea, CA) and analyzed with FlowJo (version 7.6.5, TreeStar, Ashland, OR).

The total numbers of cells were estimated from a FACS‐based cell count of single‐cell suspensions. Flowcount beads (Beckman‐Coulter) were added to the cell samples and cell counts were calculated by the following equation: viable cells × total beads/counted beads.

For staining of Ki67 and Foxp3, cells were stained with Zombie Yellow to eliminate dead cells. The cells were then pretreated with Fc Block, stained with FITC‐conjugated anti‐Thy1.1, PerCP/Cyanine5.5‐conjugated anti‐CD4, APC‐conjugated anti‐CD3, APC/Cyanine7‐conjugated anti‐CD8, PE/Dazzle594‐conjugated anti‐B220, anti‐F4/80, anti‐NK1.1 and anti‐Ly6G, followed by fixation, permeabilization and staining with PE‐conjugated anti‐Foxp3 and PE/Cyanine7‐conjugated anti‐Ki67 (Thermo Fisher Scientific) using Foxp3/Transcription Factor Staining Buffer Set (Thermo Fisher Scientific) according to the manufacturer's protocols.

For intracellular cytokine staining, cells were stimulated with 1 µg mL^−1^ hgp100 peptide in the presence of 10 µg mL^−1^ brefeldin A at 37 °C for 4 h. The cells were stained with Zombie Yellow, FITC‐conjugated anti‐Thy1.1, APC/Cyanine7‐conjugated anti‐CD8, Pacific Blue‐conjugated anti‐CD3, PE/Dazzle594‐conjugated anti‐B220, anti‐F4/80, anti‐NK1.1 and anti‐Ly6G, followed by fixation, permeabilization and staining with PE/Cyanine7‐conjugated anti‐IFNγ, PE‐conjugated anti‐TNFα and APC‐conjugated anti‐Granzyme B (BioLegend) using Intracellular Staining Fixation Buffer and Intracellular Staining Permeabilization Wash Buffer (10X) (BioLegend) according to the manufacturer's protocols.

To analyze uptake of glucose analogue, cells were stained with Zombie Yellow, APC‐conjugated anti‐Thy1.1, APC/Cyanine7‐conjugated anti‐CD8, Pacific Blue‐conjugated anti‐CD3 mAbs, PE/Dazzle594‐conjugated anti‐B220, anti‐F4/80, anti‐NK1.1 and anti‐Ly6G, followed by culture with 2‐[N‐(7‐Nitrobenz‐2‐oxa‐1,3‐diazol‐4‐yl) amino]‐ 2‐deoxy‐D‐glucose (2‐NBDG, Peptide Institute, Osaka, Japan) at 80 µm for 30 min at 37 °C in glucose‐free RPMI‐1640 containing 10% FBS.

Stained cell data were acquired on a Gallios flow cytometer (Beckman Coulter) and analyzed with FlowJo (version 7.6.5, TreeStar, Ashland, OR). Compensation was performed manually using single‐color controls for each fluorochrome. The raw flow cytometry datasets supporting this study are publicly available in Zenodo (DOI: 10.5281/zenodo.17196983, 10.5281/zenodo.1719701, 10.5281/zenodo.17197038, 10.5281/zenodo.17197107, 10.5281/zenodo.17197319, 10.5281/zenodo.17197360, 10.5281/zenodo.17197425 and 10.5281/zenodo.17197460).

### 4T1 Flow Cytometry

Orthotopic 4T1 tumors were established on female BALB/c mice (5 weeks‐old) by inoculating 4T1 cells to the left third mammary fat pad on day 0. The treatments were conducted after the average tumor volume reached 100 mm^3^ (day 10). Daily dexamethasone treatments (0.06 mg in 100 µL PBS, equivalent to 3mg/kg in a mouse with 20‐gram body weight, i.p. injection) were conducted for 6 days (day 10–15). ICB treatments were conducted on Day 12 and 15 for twice. In each ICB treatment, 200 µg anti‐PD‐1 antibody and 100 µg anti‐CTLA‐4 antibody were i.p. injected to the mice. On Day 17, the primary tumors and tumor draining lymph nodes were harvested from the mice for flow cytometry analysis.

Tumors were collected on day 17, cut into pieces and incubated in RPMI‐1640 (Nacalai Tesque) supplemented with 1% FBS, 10 mM HEPES, 0.2% collagenase (FUJIFILM Wako Pure Chemical Corporation, Osaka, Japan) and 2 KU/mL DNase I (Sigma–Aldrich) for 40 min at 37 °C. All material was passed through a 70 µm cell strainer to obtain single cell suspensions.

For staining of myeloid cells, the cells were stained with Zombie Aqua (BioLegend). The cells were then pretreated with Fc Block and stained with Pacific Blue anti‐CD45, APC/Cyanine7‐conjugated anti‐CD11c, FITC‐conjugated anti‐I‐A/I‐E, PE‐conjugated anti‐CD64, APC‐conjugated anti‐CD103, PerCP/Cyanine5.5‐conjugated anti‐CD11b, PE/Cyanine7‐conjugated anti‐Ly6C and PE/Dazzle594‐conjugated anti‐Ly6G.

For staining of Ki67 and Foxp3, cells were stained with Zombie Aqua to eliminate dead cells. The cells were then pretreated with Fc Block and stained with PE/Dazzle594‐conjugated anti‐B220, anti‐F4/80 and anti‐Ly6G, PerCP/Cyanine5.5‐conjugated anti‐CD4, APC‐conjugated anti‐CD3 and APC/Cyanine7‐conjugated anti‐CD8, followed by fixation, permeabilization and staining with PE‐conjugated anti‐Foxp3 and PE/Cyanine7‐conjugated anti‐Ki67 using Foxp3/Transcription Factor Staining Buffer Set according to the manufacturer's protocols.

For intracellular cytokine staining, cells were stimulated with 10 ng mL^−1^ phorbol 12‐myristate 13‐acetate PMA, Sigma‑Aldrich) and 1 µg mL^−1^ ionomycin (Sigma‑Aldrich) in the presence of 10 µg mL^−1^ brefeldin A (Sigma‐‑Aldrich) at 37 °C for 4 h. The cells were stained with Zombie Yellow, FITC‐conjugated anti‐CD3, APC/Cyanine7‐conjugated anti‐CD8 and Pacific Blue‐conjugated anti‐CD45, PE/Dazzle594‐conjugated anti‐B220, anti‐F4/80 and anti‐Ly6G, followed by fixation, permeabilization and staining with APC‐conjugated anti‐Granzyme B using Intracellular Staining Fixation Buffer and Intracellular Staining Permeabilization Wash Buffer (10×) according to the manufacturer's protocols.

To analyze uptake of glucose analogue, cells were stained with Zombie Yellow, APC‐conjugated anti‐CD3, APC/Cyanine7‐conjugated anti‐CD8, PerCP/Cyanine5.5‐conjugated anti‐CD4, and Pacific Blue‐conjugated anti‐CD45, PE/Dazzle594‐conjugated anti‐B220, anti‐F4/80 and anti‐Ly6G, followed by culture with 2‐NBDG at 80 µm for 30 min at 37 °C in glucose‐free RPMI‐1640 containing 10% FBS.

Stained cell data were acquired on a Gallios flow cytometer or CytoFlex (Beckman‐Coulter) and analyzed with FlowJo. Compensation was performed manually using single‐color controls for each fluorochrome. The raw flow cytometry datasets supporting this study are publicly available in Zenodo (DOI: 10.5281/zenodo.17196572, 10.5281/zenodo.17196667, 10.5281/zenodo.17196691, 10.5281/zenodo.17196740 and 10.5281/zenodo.17196945).

### MRI Measurements

MRI experiments were performed using a preclinical 7 Tesla 20‐cm bore MRI system (BioSpec, Avance‐III system, Bruker Biospin, Ettlingen, Germany) with a highly sensitive cryogenic RF coil (2ch, Cryoprobe, transmit and reception, Bruker Biospin). For in vivo MRI studies, 3 mg kg^−1^ dexamethasone was administered by i.p. injection once a day for five days starting from day 7 to day 11 post‐implantation and before tumor excision. MR images at day 11 post‐implantation were acquired with PEGylated gadolinium (Gd)‐DOTA‐dendron‐liposome contrast agent (Cat. #KH16000590, DS pharma biomedical, Osaka, Japan) administration intravenously.

During the in vivo MRI experiments, mice were anesthetized with 2.0% isoflurane gas (Escain, Mylan, PA, USA) and a 1:5 = O_2_: room‐air mixture. Rectal temperature was measured via an optical fiber thermometer (FOT‐L, FISO Technology, Quebec City, QC, Canada) inserted into the rectum, and maintained at 36.5 ± 0.5 °C via a warm water pad and warm air heating systems. Body temperature and breathing were monitored via a Biopac system (BIOPAC Systems, Goleta, CA, USA). MR angiography (MRA) for visualizing vascular structures in the tumors was acquired using a “fast low angle shot (FLASH)” sequence with the following imaging parameters: repetition time (TR)/echo time (TE) = 15.0/2.5 ms, field of view (FOV) = 12.8 mm^3^, matrix size = 256 × 256 × 256, flip angle = 20°, number of excitations (NEX) = 3, and scan time = 36 min 51 sec. The voxel size was 50 µm^3^ isotropic. Maximum intensity projection (MIP) mapping was calculated using the MRA data. To calculate the tumoral vessel volume, MRA images were binarized using a 50% intensity threshold, and the total area of the enhanced regions of the binary map was measured using ITK‐SNAP version 3.2 (University of Pennsylvania and University of Utah).

### Primary Tumor Efficacy Study

For the T cell depletion studies, orthotopic 4T1‐HA tumors were established on female BALB/c mice (5 weeks‐old) by inoculating 4T1‐HA cells to the left third mammary fat pad on day 0. The treatments were conducted after the average tumor volume reached 100 mm^3^ (day 9). For depleting the immune cells, mice were i.p. injected with 200 µg anti‐CD4 (BioXcell, clone GK1.5), anti‐CD8 (BioXcell, cline 53‐6.7) or anti‐asialo GM1 (FUJIFILM Wako Pure Chemical Corporation, Cat. No 014‐09801) antibodies every two days for a total of three treatments (day 9, 11 and 13). The daily dexamethasone treatments (0.06 mg in 100 µL PBS, equivalent to 3mg/kg in a mouse with 20‐gram body weight, i.p. injection) were started from Day 10 and performed until the end of the experiment. Tumor volumes of individual mouse were monitored during the whole experiment.

For primary tumor efficacy studies, treatment of mice bearing 4T1‐HA tumors was initiated 7–8 days after inoculation. Tumors were size‐ and time‐matched, and the average volume for the groups at treatment initiation varied between 104 and 107 mm^3^. Dexamethasone (0.06 mg in 100 µL PBS, equivalent to 3mg/kg in a mouse with 20‐gram body weight) was administered by i.p. injection daily from day 8 until day 13 post‐inoculation. The ICB cocktail of anti‐PD‐1 (200 µg in 100 µL PBS, RMP 1‐14 eBioscience) and anti‐CTLA‐4 (100 µg in 100 µL PBS, 9D9 BioXcell) was administered i.p. on days 10 and 13. Tumor volume was measured with calipers and the experimenter was blind of the treatment groups. On day 15, mice were euthanized and tumors and spleens were weighed.

In E0771, treated was initiated 11 days after inoculation, and the average volume for the groups at treatment initiation varied between 95 and 96 mm^3^. Dexamethasone (0.06 mg in 100 µL PBS, equivalent to 3mg/kg in a mouse with 20‐gram body weight) was administered by i.p. injection daily from day 11 until day 16 post inoculation. The ICB cocktail of anti‐PD‐1 (200 µg in 100 µL PBS, RMP 1‐14 eBioscience) and anti‐CTLA‐4 (100 µg in 100 µL PBS, 9D9 BioXcell) was administered i.p. on days 13 and 16. On day 19, mice were euthanized, and tumors and spleens were weighed.

For all tumor studies, mice were randomized by tumor volume and number of days since tumor inoculation. Researchers performing tumor volume measurement before randomization and on‐study were blinded from treatment group.

### In Vitro Cytotoxicity

The in vitro cytotoxicity of t dexamethasone was examined against 4T1‐HA. Cancer cells were plated into flat‐bottomed, 96‐ well plates at 5 × 10^3^ cells per well. Four measurements were performed per dose of dexamethasone. They were treated by continuous exposure to various concentrations of dexamethasone in a final volume of 100 µL. Plates were incubated for 48 h at 37 °C in a humidified atmosphere with 5% CO_2_, and cell viability was determined by CCK‐8 assay. We used 1mM (1000 µm) highest concentration, and then 2 times dilution.

### 4T1‐HA Flow Cytometry

Tumors were treated as in the primary tumor efficacy study. On day 15 post‐inoculation, tumors were collected, cut into pieces, and transferred to gentle‐MACS C Tubes containing an enzyme mix (Miltenyi biotech, Bergish Gladbach, Germany) and passed through a 70 µm cell strainer (Fisher Scientific, Hampton, NH) to obtain tumor‐infiltrating cells. After eliminating dead cells with Zombie Yellow, HA‐specific T cells were detected by T‐Select H‐2Kd Influenza HA Tetramer‐IYSTVASSL‐PE (MBL, Nagoya, Japan) with APC‐Cy7‐conjugated anti‐CD45, FITC‐conjugated anti‐CD8 and PerCP/Cy5.5‐conjugated anti‐CD4 (BioLegend). T‐Select H‐2Kd EGFP Tetramer‐HYLSTQSAL‐PE (MBL) was used as control. Cells were stimulated with 1µg/mL of HA peptide (HA 533‐541, IYSTVASSL) for 4 h; IFNγ‐producing cells were detected by intracellular IFNγ staining method using Intraprep Permeabilization Reagent (Beckman‐Coulter) according to the manufacturer's protocols with APC‐conjugated anti‐IFNγ antibody or isotype control (Rat IgG2a, κ, Biolegend) at 4 °C for 30 min. Stained cell data were acquired on a Gallios flow cytometer (Beckman Coulter) and analyzed with FlowJo (version 7.6.5, TreeStar, Ashland, OR). Compensation was performed manually using single‐color controls for each fluorochrome. The raw flow cytometry datasets supporting this study are publicly available in Zenodo (DOI: 10.5281/zenodo.17196296).

### Fluorescent Immunohistochemistry

For histological studies, dexamethasone (3 mg kg^−1^ or 30 mg kg^−1^) or equal volume PBS was administered by i.p. injection once a day for four days starting from day 7 to day 11 post‐implantation and before tumor excision. 4T1 breast tumors were removed, incubated with 4% paraformaldehyde for 40 min and washed twice for 10 min with 1× PBS. Fixed tissues were embedded in optimal cutting temperature compound (OCT) in cryomolds (Tissue‐Tek) and frozen completely at −20 °C. Transverse 20 µm‐thick tumor sections were produced using the Tissue‐Tek Cryo3 (SAKURA). Positively charged HistoBond microscope slides (Marienfeld) were used to bound four tissue sections per tumor. Tumor sections were then incubated in blocking solution (10% fetal Bovine Serum, 3% Donkey Serum, 1× PBS) for 2 h and immunostained with the following primary antibodies; rat anti‐CD3 (17A2, BioLegend 1:100), hamster anti‐CD11c (HL3, BD Pharmingen 1:100), rat anti‐CD206 (MR5D3, BIO‐RAD 1:50) and rat anti‐F4/80 (A3‐1, BIO‐RAD 1:50) overnight at 4 °C. Secondary antibodies against hamster [Alexa Fluor‐488 anti‐hamster IgG (H+L) (ab173003, Abcam 1:200)] or rat [Alexa Fluor‐647 anti‐rat IgG (H+L) (A21247, Invitrogen 1:400)] were used. All samples were incubated in secondary antibody solution, including DAPI (Sigma, 1:100 of 1mg mL^−1^ stock) for 2 h at room temperature in the dark. Sections were mounted on microscope slides using the ProLong™ Gold Antifade Mountant (Invitrogen) and covered with a glass coverslip. Images of stained tumors sections were acquired at constant microscope settings that avoided saturation at 10× magnification using an Olympus BX53 fluorescence microscope. The pixel size of the images was 1.98 pixel/µm.

### RNA Isolation, cDNA Synthesis and Real‐Time PCR

Total RNA was isolated from breast tumors according to the standard Trizol‐based protocol (Invitrogen) and cDNA synthesis was performed using reverse transcriptase III (RT‐III) enzyme and random hexamers (Invitrogen). Real‐time polymerase chain reaction (PCR) was performed using Sybr Fast Universal Master Mix (KAPA). The specific mouse primers used for gene expression analysis of 4T1 tumors for *Cxcl13* are (F 5’‐TCGTGCCAAATGGTTACAAA‐3’, R 5’‐GCTTGGGGAGTTGAAGACAG‐3’). The primers used for β‐actin are F 5’‐GACGGCCAGGTCATCACTAT‐3’ and R 5’‐AAGGAAGGCTGGAAAAGAGC‐3’. Reactions were performed using a CFX‐96 real‐time PCR detection system (Biorad) at the following conditions: 95 °C for 2 min, 95 °C for 2 s, 60 °C for 20 s, 60 °C for 1 s, steps 2–4 for 39 cycles. Real‐time PCR analysis and calculation of changes in gene expression between compared groups were performed using the ΔΔCt method. Relative gene expression was normalized based on the expression of β‐actin.

### T Cell Distribution Analysis of Fluorescent Immunohistochemistry

Distances between CD3^+^ T cells and the corresponding nearest αSMA^+^ area were calculated using custom MATLAB scripts with built‐in image processing functions.^[^ ^26^, ^28^, ^40^ ^]^ CD3^+^ T cells were determined automatically based on signal intensity, size, and morphology thresholds and αSMA^+^ area was similarly determined based on an intensity threshold. The thresholds were chosen manually by reviewing the attributes of all images, and the same thresholds were used for all images. Thresholds were determined using sections stained with secondary antibodies only to avoid background florescence and non‐specific staining. Researchers performing image analysis were blinded from the treatment group.

To measure the distance, the centroids of CD3^+^ T cells were determined, and each cell's distance to the nearest αSMA^+^ area was measured by finding the distance between the centroids and the nearest αSMA^+^ pixels. Also, images were divided into two groups. One group contains images that represent αSMA‐rich tissue, and the other group contains images that are αSMA‐poor. The average αSMA^+^ area fraction was calculated and used as the parameter to determine which images displayed a high or low αSMA^+^ area fraction. Quality control on images was performed, as before,^[^ ^28^ ^]^ first by ensuring all images in the analysis had the tissue in the focal plane. Imaging occurred in regions free of folds, tears, and external artifacts. Consistent microscope settings that avoided saturation. Automatically segmented images were checked to ensure the cell boundaries of the image matched the mask. The distanced between cells in representative images was measured manually in ImageJ to ensure correlation with the automated measurement.

To assess the heterogeneity of T cell and macrophage distribution in tumors, images of CD3, F480 and CD11c were segmented as above. Images were broken into square grids with side length 100 pixels, as in our previous work.^[^ ^28^ ^]^ The number of grids with at least one centroid of a cell of interest and half of the grid pixels with intensity in the channel of interest greater than the threshold determined using images of tissue slides stained without the primary antibody. If T cells or macrophages were excluded, they would remain in fewer grids. If T cells or macrophages could penetrate, they would be in more grids.

### Spontaneous Metastasis Model Survival Studies

For the metastasis survival studies, 4T1 and E0771 mice were prepared as described above. We performed an a priori power analysis using a two‐tailed significance level of alpha = 0.05 and 85% power to observe an 80% increase in survival and assumed a standard deviation of 40%, based on previous studies. When 4T1 tumors reached ≈300 mm^3^ and E0771 tumors reached 1000 mm^3^ the primary tumors were removed, as described previously.^[^ ^15^, ^26^, ^27^ ^]^ The 4T1 bearing mice were given two days of rest while the E0771 were given four days of rest before treatment initiation. Mice were size‐matched based on tumor volume at resection into treatment groups and dexamethasone was administered on day 0 (day of treatment initiation), continuing daily until day 5. The ICB cocktail was administered on days 2 and 5. Mice were observed and weighed three or four times per week and euthanized based on ethical guidelines. Surviving mice were rechallenged on day 100 after primary tumor resection and compared against tumor‐naïve age‐matched controls.

### Statistical Analysis

The data are presented as means with error bars representing the standard error of the mean. Groups were compared using one‐way ANOVA with comparisons between groups tested using Tukey's method, except in the metastasis survival study, where a log‐rank test was used and *P* values were adjusted using Holm‐Bonferroni correction. The sample size for each study is reported throughout the text. GraphPad Prism 9 software was used for statistical analysis.

## Conflict of Interest

The authors declare no conflict of interest.

## Author Contributions

J.D.M, K.N, and M.P. contributed equally to this work. J.D.M, K.N. and M.P. performed all the experiments and analyzed the data. A.H., T.T.K, P.C. and C.S. analyzed the immune cells in tumors and organs. F.M., P.P., C.V. and P.C. evaluated the antitumor efficacy. M.R.M. analyzed the images. A.S., N.N. and I.A. performed the 3D MRI angiography. K.T. provided cell lines, J.D.M. and H.C. wrote the manuscript. K.N., M.P., K.K., K.K and T.F. edited the manuscript. T.F., K.K. and H.C. supervised the project.

## Supporting information



Supporting Information

Supplemental Movie 1

Supplemental Movie 2

## Data Availability

The data that support the findings of this study are available from the corresponding author upon reasonable request.
