# Supplementary material for: Antiemesis Corticosteroids Potentiate Checkpoint Blockade Efficacy by Normalizing the Immune Microenvironment in Metastatic Murine Breast Cancer
Source: Adv Sci (Weinh). 2025 Nov 28;13(14):e14261. doi: 10.1002/advs.202514261 (PMC12970202; doi:10.1002/advs.202514261)
Supplement: Supplementary file 1 — Supporting Information [file ADVS-13-e14261-s001.docx]

Supporting Information

**Antiemesis Corticosteroids Potentiate Checkpoint Blockade Efficacy by Normalizing the Immune Microenvironment in Metastatic Murine Breast Cancer**

*John D. Martin^1,#^, Koji Nagaoka^2,3#^, Myrofora Panagi^4,#^, Akihiro Hosoi^2^, Fotios Mpkeris^4,5^,*

*Pengwen Chen^1^, Thahomina T. Khan^1^, Margaret R. Martin^1^, Changbo Sun^2^, Chrysovalantis*

*Voutouri^4^, Maria Louca^4^, Panagiotis Papageorgis^4,7^, Akira Sumiyoshi^6^, Nobuhiro Nitta^6^, Kazuyoshi Takeda^8,9^, Ichio Aoki^6^, Kazunori Kataoka^10^, Triantafyllos Stylianopoulos^4*^, Kazuhiro Kakimi^2,3*^, Horacio Cabral^1*^*

1. *Department of Bioengineering, Graduate School of Engineering, The University of Tokyo, Bunkyo, Tokyo, 113-8656, Japan.*
2. *Department of Immunotherapeutics, The University of Tokyo Hospital, Bunkyo, Tokyo, 1138656, Japan.*
3. *Department of Immunology, School of Medicine, Kindai University, Osakasayama, Osaka, 589-8511, Japan.*
4. *Cancer Biophysics Laboratory, Department of Mechanical and Manufacturing Engineering, University of Cyprus, Nicosia, 1678, Cyprus.*
5. *Cancer Genetics, Therapeutics & Ultrastructural Pathology Department, The Cyprus Institute of Neurology & Genetics, Nicosia, 2371, Cyprus.*
6. *National Institute of Radiological Sciences, Japan Agency for Quantum and Radiological Science and Technology, Anagawa 4-9-1, Inage, Chiba 263-8555, Japan.*
7. *Department of Life Sciences, Program in Biological Sciences, European University Cyprus, Nicosia, 1516, Cyprus.*
8. *Division of Cell Biology, Biomedical Research Center, Juntendo University, Bunkyo-ku, Tokyo, 113-8421 Japan.*
9. *Department of Biofunctional Microbiota, Graduate School of Medicine, Juntendo University, Bunkyo-ku, Tokyo, 113-8421, Japan.*
10. *Innovation Center of NanoMedicine, Kawasaki Institute of Industrial Promotion, Kawasaki, Kanagawa, 210-0821, Japan.*

^#^ *These authors contributed equally.*

** Correspondence to: stylianopoulos.triantafyllos@ucy.ac.cy (TS),* *kakimi@med.kindai.ac.jp (KK), horacio@bmw.t.u-tokyo.ac.jp (HC)*

**Supplementary Table 1** **– Dexamethasone dose schedule**. Dose schedule (timing in the header) of the current study in mice (top row) compared to an antiemesis regimen in a clinical trial (NCT02043288) converted from human to mouse doses by body surface area (bottom row). The total dose of dexamethasone is in the right column.

| Time relative to treatment initiation | -48  [Hours] | -24 | -12 | -6 | 0 | 12 | 24 | 36 | 48 | Total dose |
| --- | --- | --- | --- | --- | --- | --- | --- | --- | --- | --- |
| Current study dose of dexamethasone | 3  [mg/kg] | 3 |  |  | 3 |  | * |  |  | 9 |
| Clinical protocol of antiemesis dexamethasone |  |  | 4.11  [mg/kg] | 4.11 |  | 0.82 | 0.82 | 0.82 | 0.82 | 11.50 |

*If another cycle is initiated, 3mg/kg would be administered at this time to begin the next cycle.

**Supplementary Table 2** **– Antibodies used for flow cytometry**.


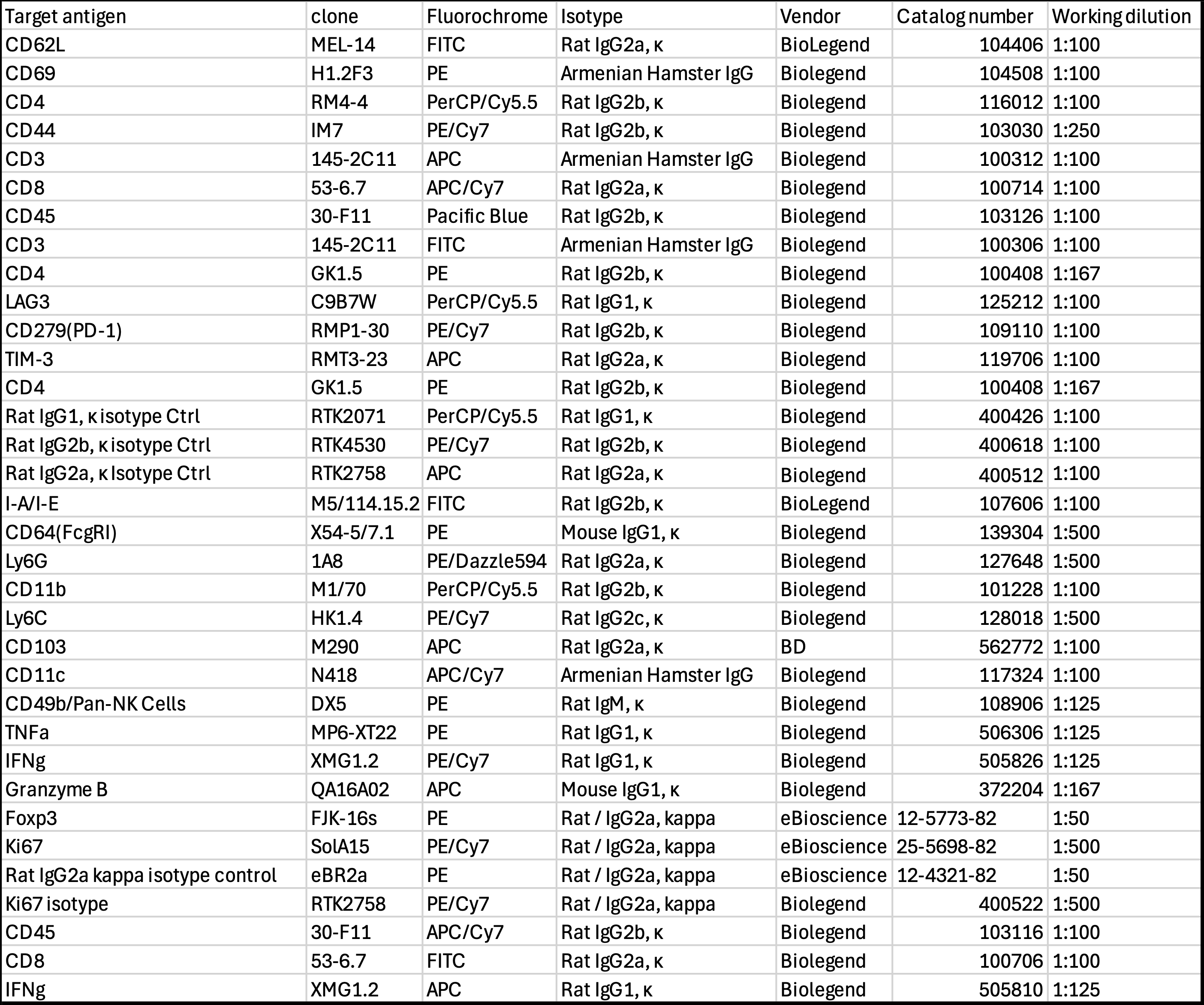


**
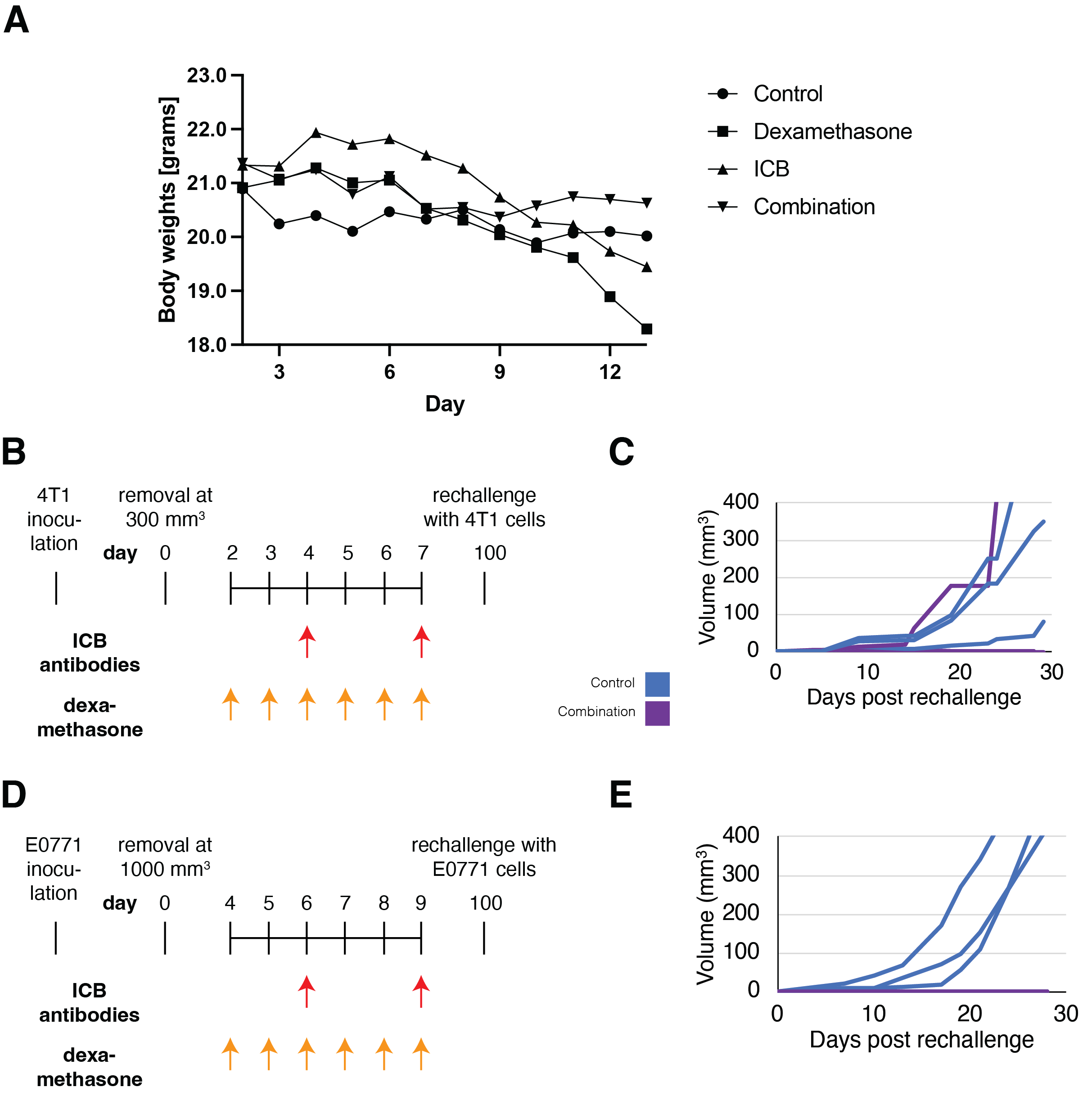
**

**Supplementary Figure 1. Mice cured after dexamethasone and ICB combination resist rechallenge.** (**A**) The mice were inoculated with 4T1 cells and their tumors were removed when they reached 300 mm^3^. The mice were treated with dexamethasone daily from day 2 until day 7 and ICB cocktail on days 4 and 7. Average body weights of the mice over time while on treatment. The combination treatment was tolerated. (**B**) On day 100 surviving mice were rechallenged. (**C**) Surviving mice were rechallenged with cancer cells and the tumor growth was compared to tumors in healthy mice. While all three healthy mice developed tumors, only one of three surviving mice developed a tumor. (**D**) The mice were inoculated with E0771 cells and their tumors were removed when they reached 1000 mm^3^. The mice were treated with dexamethasone daily from day 4 until day 9 and ICB cocktail on days 6 and 9 and on day 100 surviving mice were rechallenged. (**E**) Surviving mice were rechallenged with tumors and their growth was compared to tumors in healthy mice. While all three healthy mice developed tumors, one of three surviving mice died without a tumor one week after rechallenge and the other two did not develop tumors.


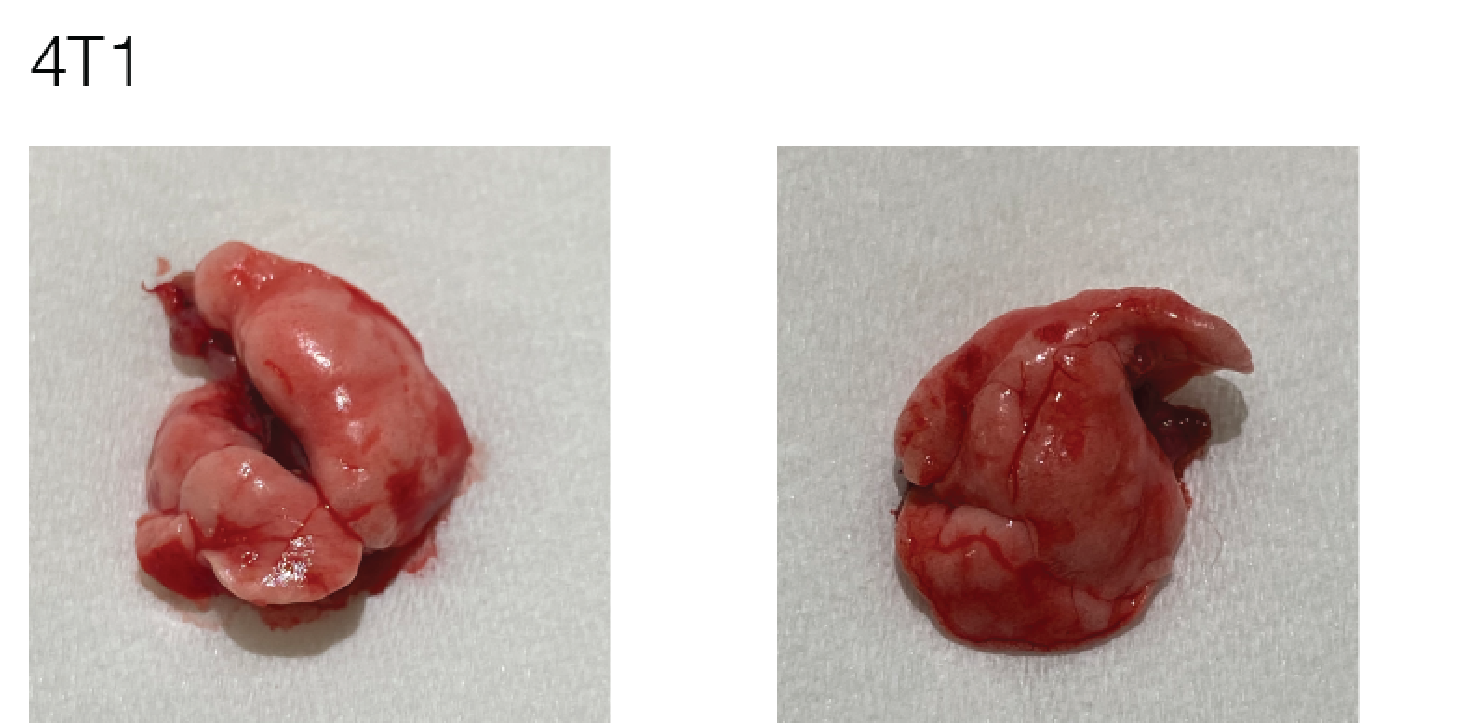


**Supplementary Figure 2. Lungs of mice after rechallenge lack macroscopic metastases.** Images of lungs extracted from the two surviving mice that resisted rechallenge with 4T1 cells.


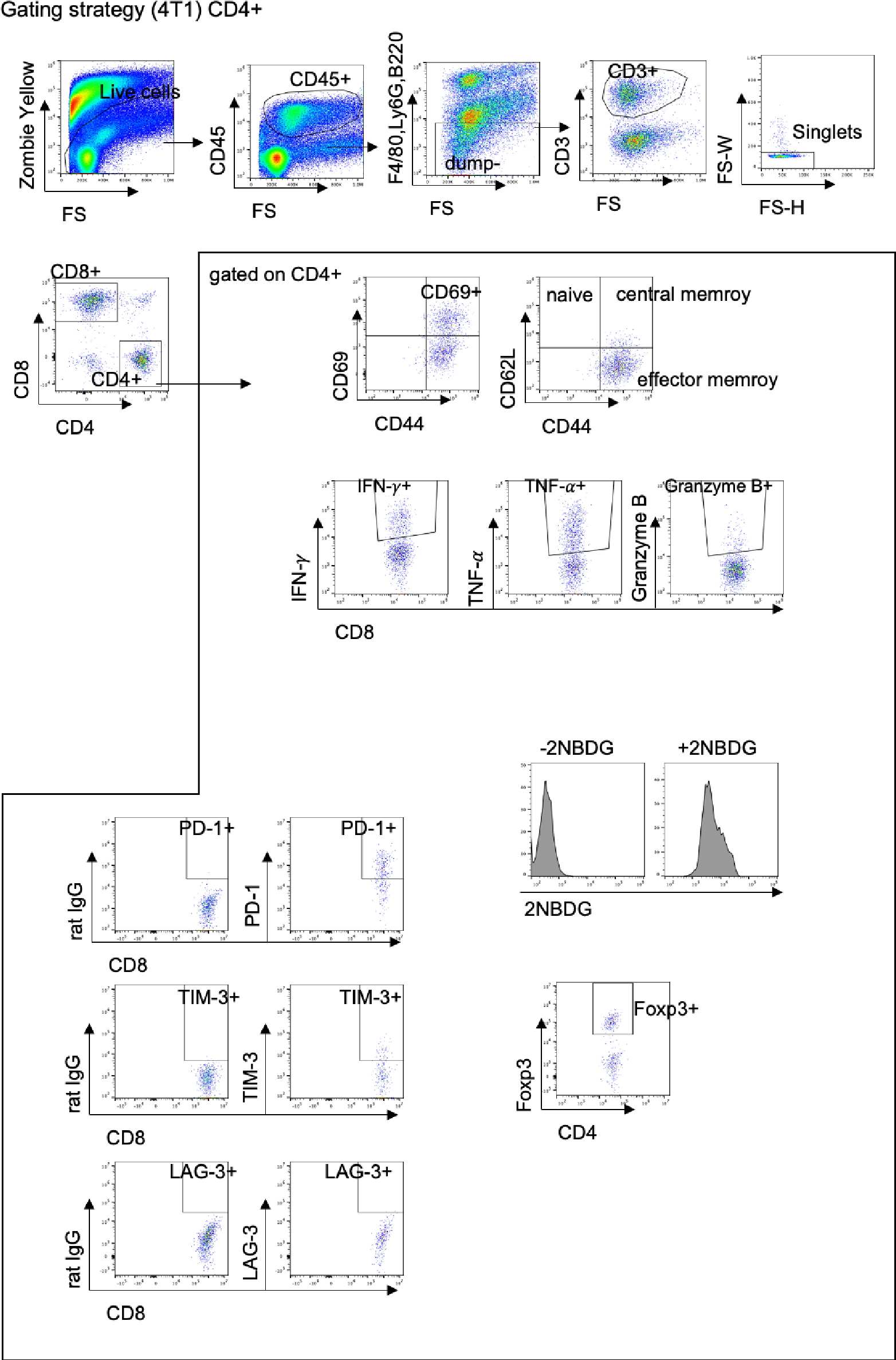


**Supplementary Figure 3. Flow cytometry gating of CD4+ T cells in 4T1 experiments.** Tumor-infiltrating lymphocytes were detected as CD3^+^CD4^+^ cells in the CD45^+^ population after myeloid cell removal. Differentiation into regulatory T cell phenotype, activation, memory, immune checkpoint expression, intracellular cytokine expression, and glucose uptake of T cells were detected.


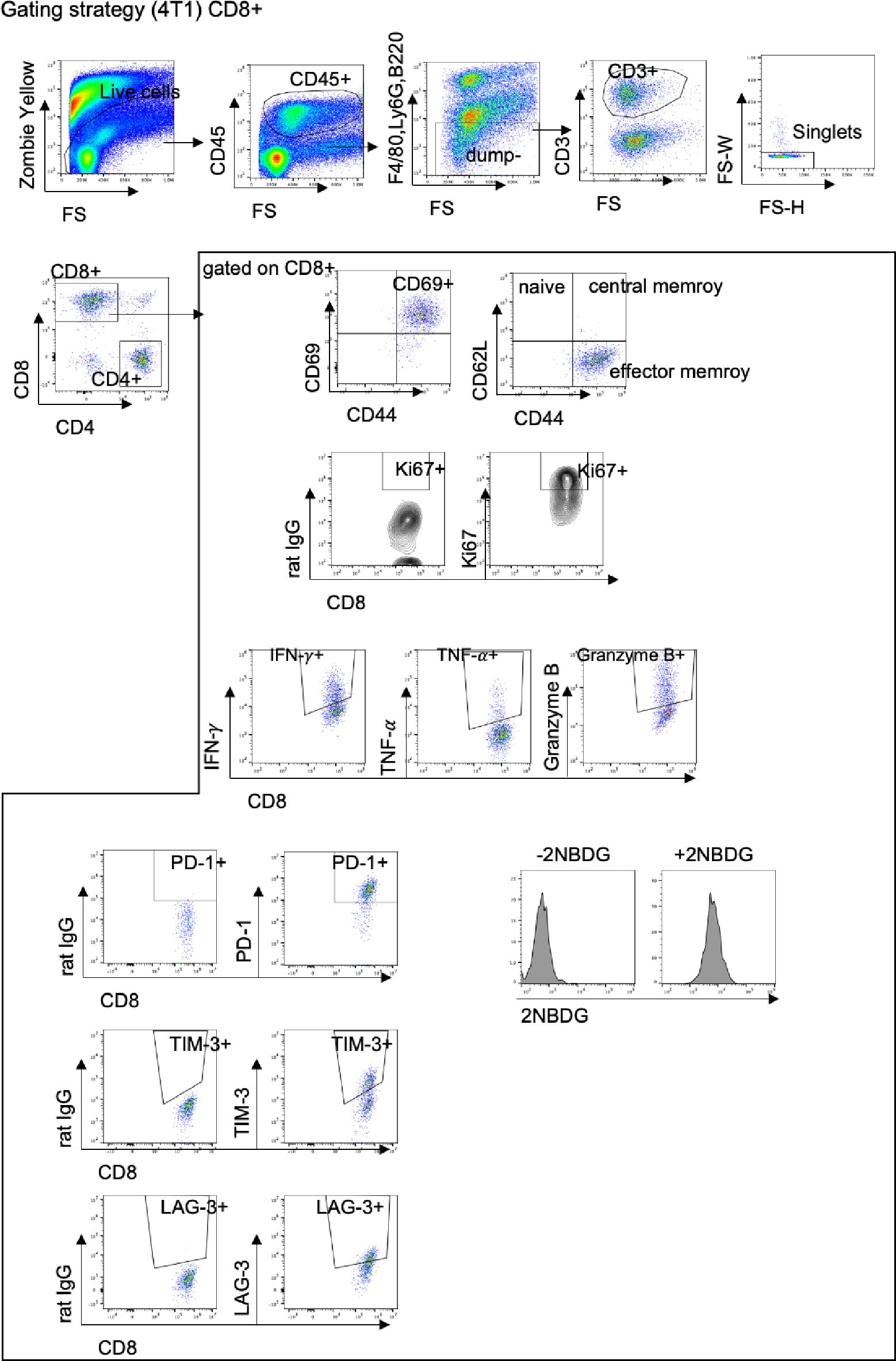


**Supplementary Figure 4. Flow cytometry gating of CD8+ T cells in 4T1 experiments.** Tumor-infiltrating lymphocytes were detected as CD3^+^CD8^+^ in the CD45^+^ population after myeloid cell removal. Proliferation, activation, memory, immune checkpoint expression, intracellular cytokine expression, and glucose uptake of T cells were detected.


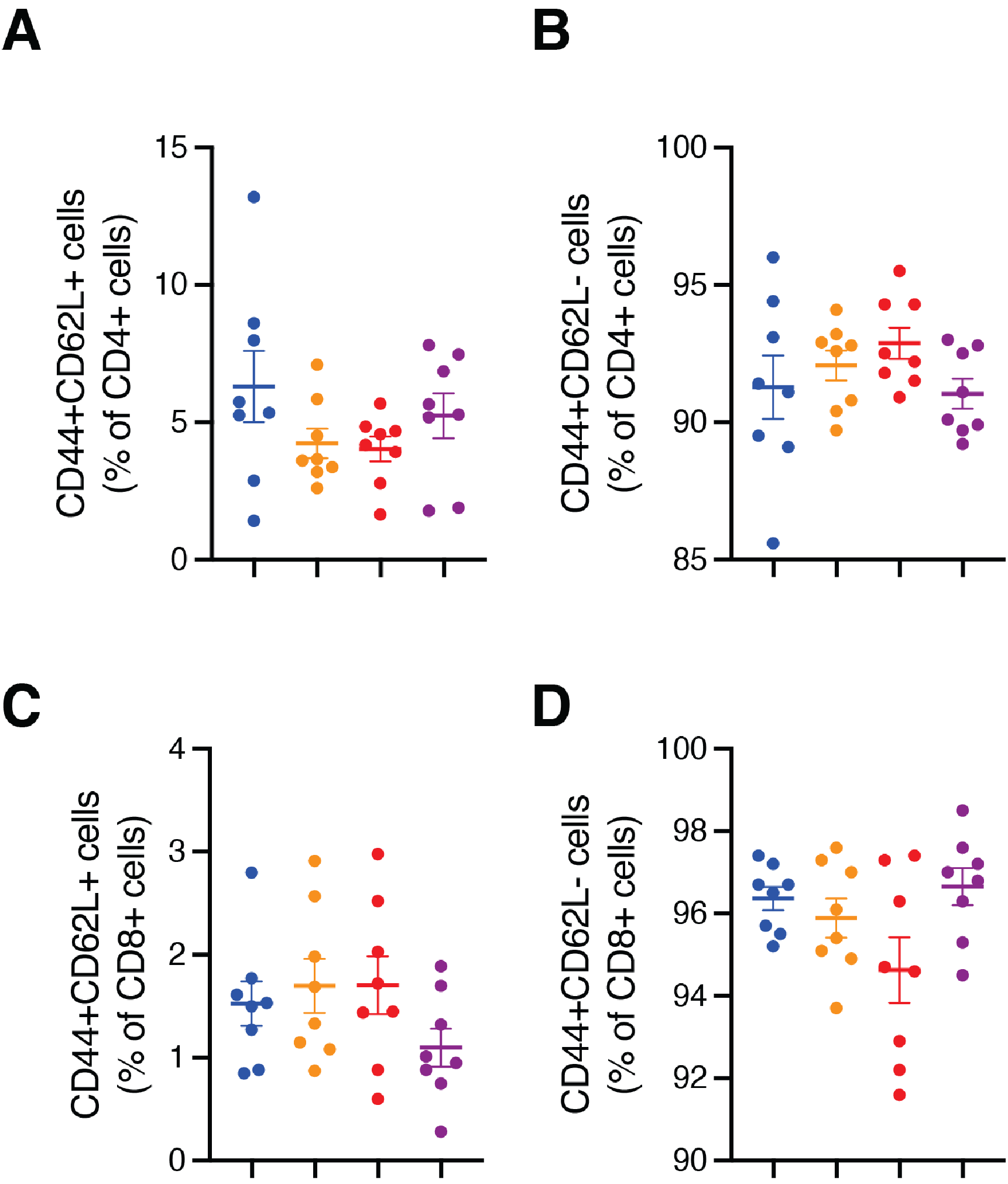


**Supplementary Figure 5. Dexamethasone does not affect fraction of effector and central memory CD4+ and CD8+ T cells in 4T1 tumors**. (**A**) The fraction of central memory (CD44+CD62L+) CD4+ T cells. (**B**) The fraction of effector memory (CD44+CD62L-) CD4+ T cells. (**C**) The fraction of CD44+CD62L+CD8+ T cells. (**D**) The fraction of CD44+CD62LCD8+ T cells. For all graphs, n = 8 mice per group and data plotted as average +/- standard error of the mean. Each dot represents one mouse. Statistical test by one-way ANOVA with Holm-Šídák’s correction (**** denotes P<0.0001, *** denotes P<0.001, ** denotes P<0.01, and * denotes *P* < 0.05).


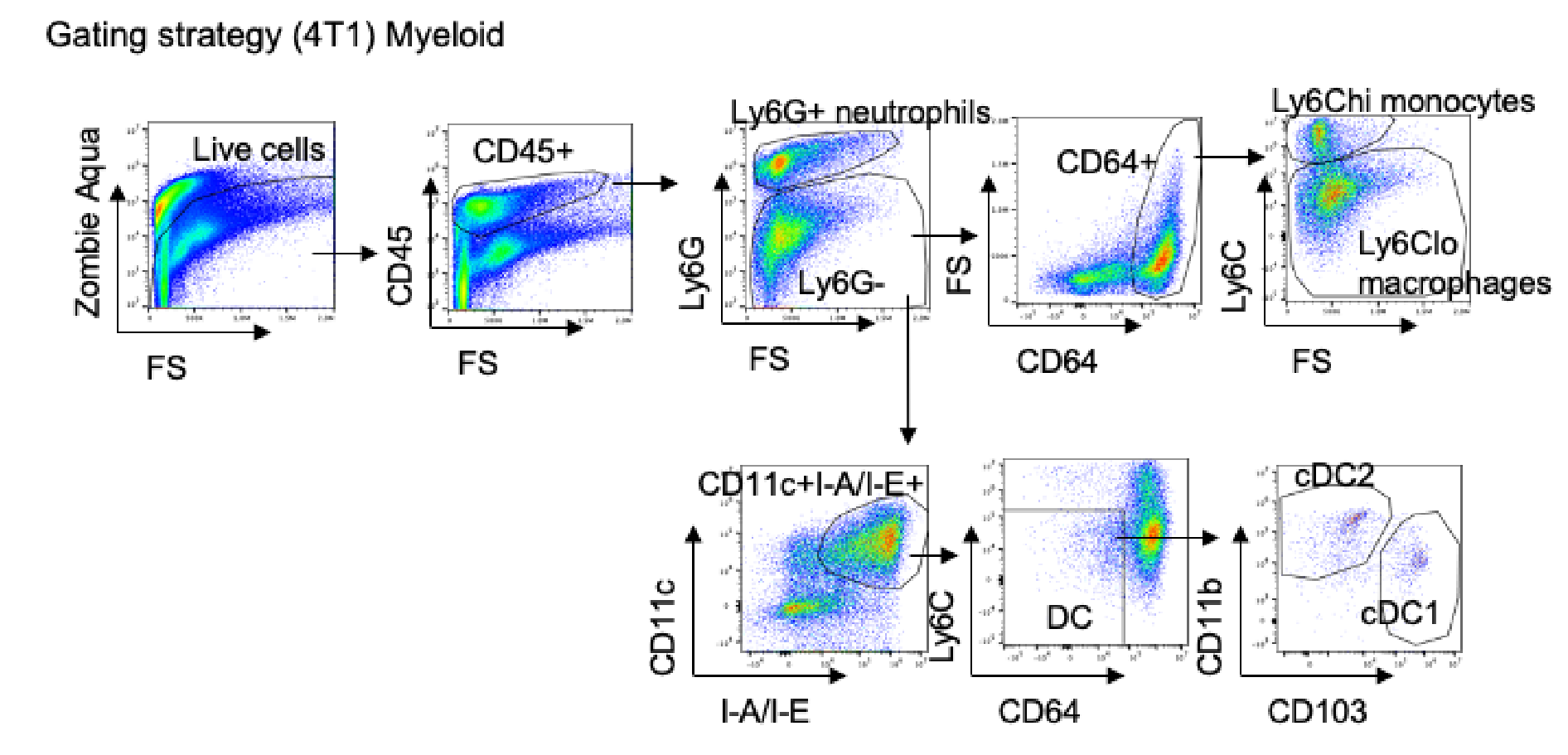


**Supplementary Figure 6. Flow cytometry gating of myeloid cells in 4T1 experiments.** Leukocytes were detected as CD45^+^ cells. Ly6G high monocytes and Ly6G low macrophages, and cDC1 and cDC2 dendritic cells were detected using CD64, CD11c, CD11b, and CD103.


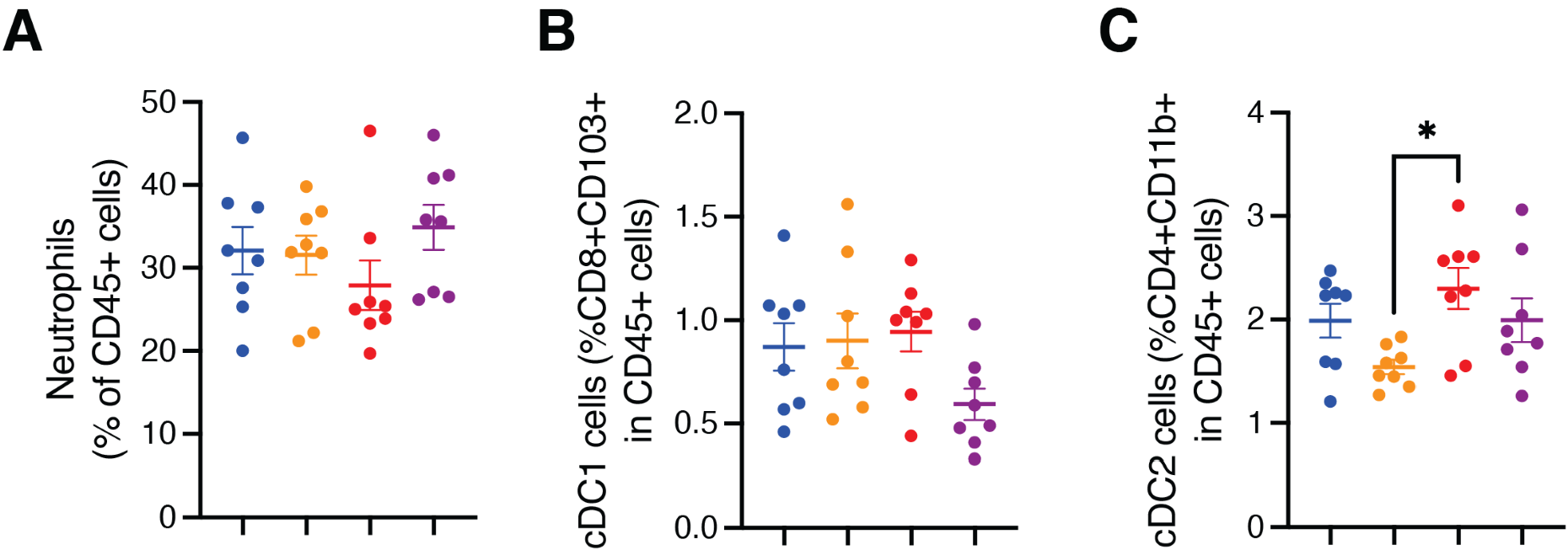


**Supplementary Figure 7. Dexamethasone does not decrease myeloid cells in untreated and ICB-treated tumors 4T1 tumors**. (**A**) The fraction of neutrophils (Ly6G+) as a percent of CD45+ leukocytes. (**B**) The fraction of cDC1 cells (CD8+CD103+) as a percent of CD45+ leukocytes. (**C**) The fraction of cDC1 cells (CD4+CD11b+) as a percent of CD45+ leukocytes. For all graphs, n = 8 mice per group and data plotted as average +/- standard error of the mean. Each dot represents one mouse. Statistical test by one-way ANOVA with Holm-Šídák’s correction (**** denotes P<0.0001, *** denotes P<0.001, ** denotes P<0.01, and * denotes *P* < 0.05).


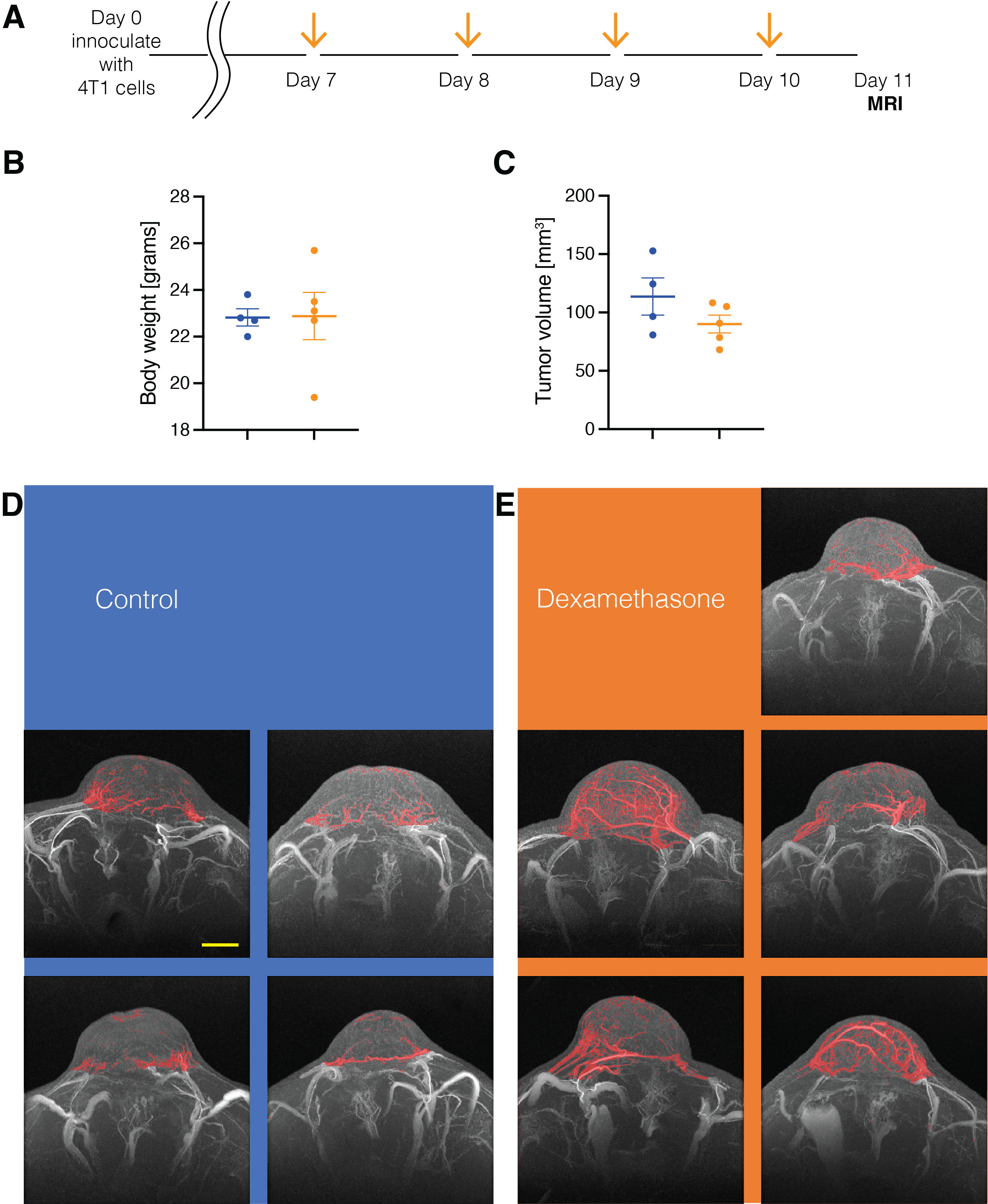


**Supplementary Figure 8. Daily dexamethasone treatment increases perfusion in 4T1 tumors without affecting mouse body weight or tumor volume.** (**A**) Experimental scheme for magnetic resonance imaging (MRI) assessment for perfused arterial and venule volume in 4T1 tumors. Mice bearing 4T1 tumors were treated with 3 mg/kg dexamethasone daily. (**B**) Body weight of mice on day 11. n = 4-5 mice (**C**) Tumor volume on day 11. n = 4-5 mice (**D**) Tumor angiographies on day 11 of tumors from the four control-treated mice in the study. Tumor vessels are indicated by the red color. (**E**) Tumor angiographies on day 11 of tumors from the five dexamethasone-treated mice in the study. Groups were compared with unpaired t tests. Data plotted as average +/- standard error of the mean. Each dot represents one mouse. Yellow scale bar indicates 1 mm.


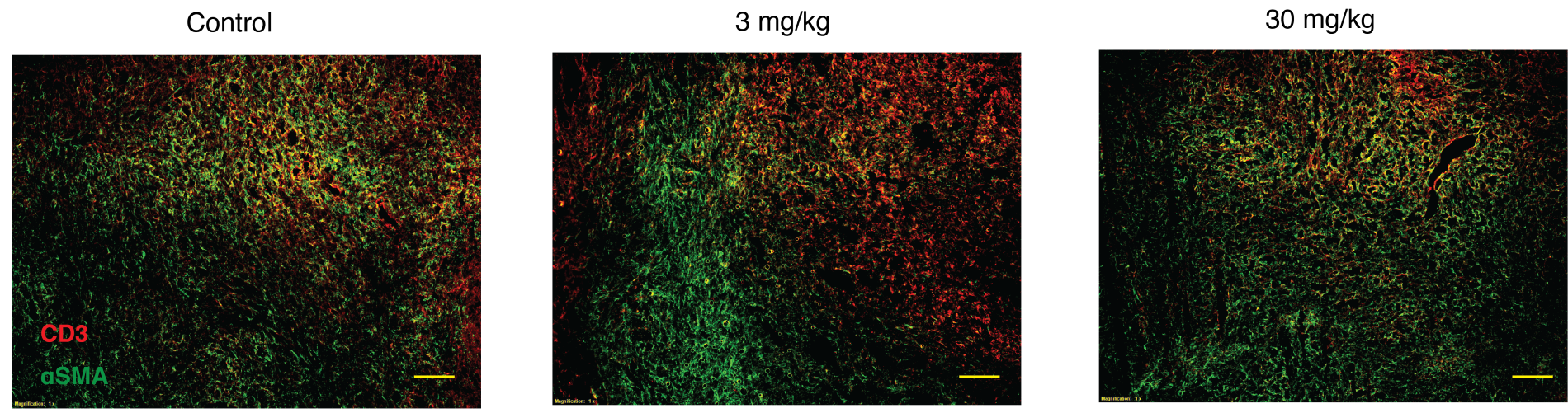


**Supplementary Figure 9. Daily dexamethasone treatment improves T cell spatial distribution in 4T1 tumors.** Representative immunofluorescence images of CD3 (red) and aSMA (green) stained 4T1 tumor tissue. Yellow scale bar indicates 0.1 mm.


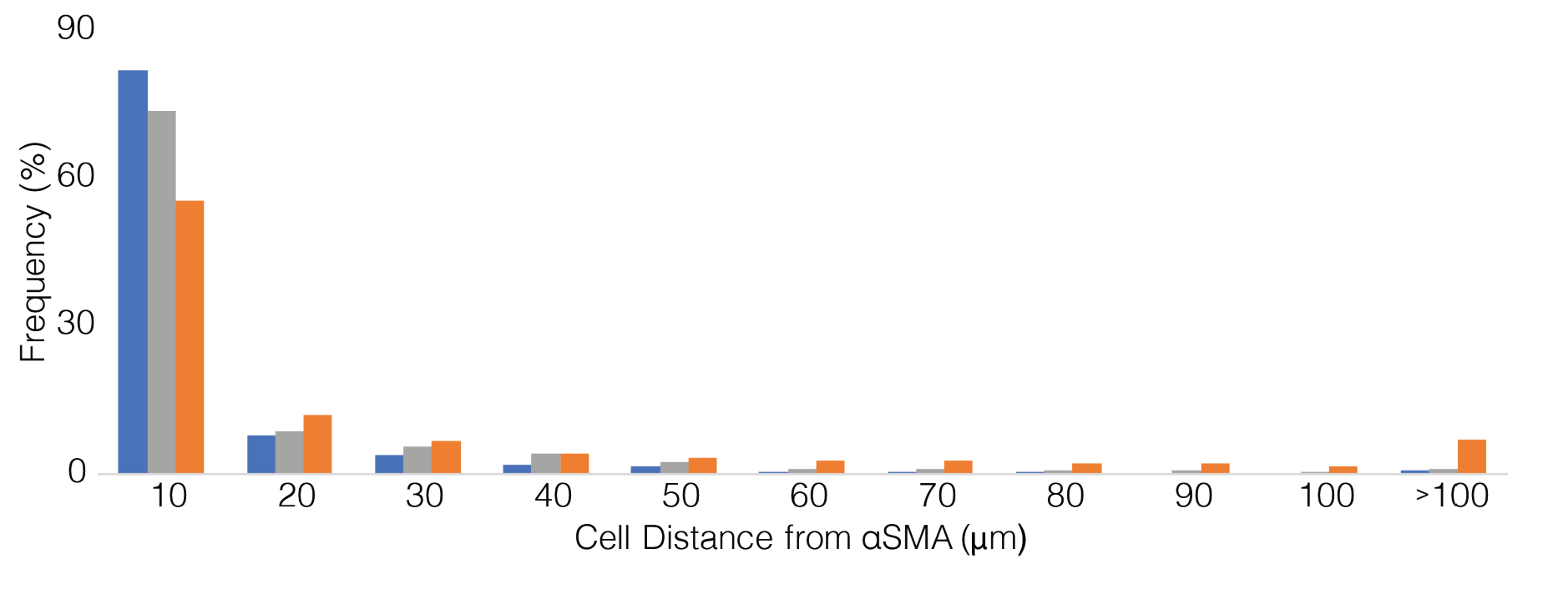


**Supplementary Figure 10. Daily dexamethasone treatment improves T cell spatial distribution in 4T1 tumors.**

Histogram of the distances between CD3+ T cells and aSMA+ areas quantified from immunofluorescence staining of mice treated with control (blue), 3 mg/kg dexamethasone (orange) and 30 mg/kg dexamethasone (gray). n = 3 mice per group, N = 8 – 12 fields.


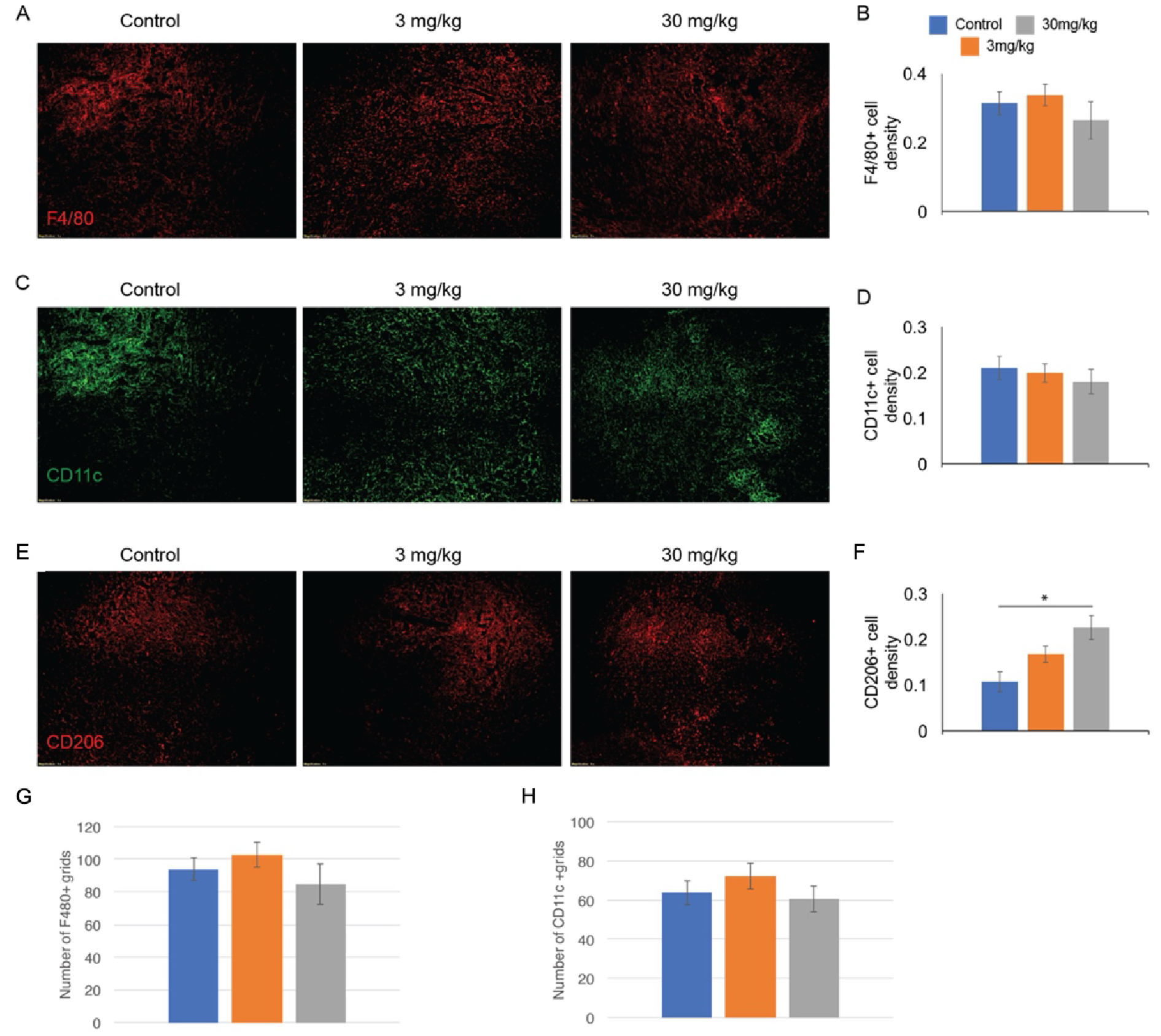


**Supplementary Figure 11. Dexamethasone does not alter TAM polarization or density**. (**A**) Representative images of 4T1 murine breast cancer stained for F4/80 (red) from control (left panel), TME-normalizing 3 mg/kg dexamethasone (center panel) and TME-depleting 30 mg/kg dexamethasone (right panel) treated mice. (**B**) Quantification of F4/80+ cell density assessed histologically. n = 3 mice, N = 9-12 images. (**C**) Representative images of 4T1 murine breast cancer stained for CD11c (green) of control (left panel), TME-normalizing 3 mg/kg dexamethasone (center panel) and TME-depleting 30 mg/kg dexamethasone (right panel). (**D**) Quantification of CD11c+ cell density assessed histologically. n = 3 mice, N = 9-12 images. (**E**) Representative images of 4T1 murine breast cancer stained for CD206 (red) from control (left panel), TME-normalizing 3 mg/kg dexamethasone (center panel) and TME-depleting 30 mg/kg dexamethasone (right panel) treated mice. (**F**) Quantification of CD206+ cell density assessed histologically. TME-depleting 30 mg/kg dexamethasone increased the density of these immunosuppressive cells (*P* < 0.05, Student’s T-test). n = 3 mice, N = 9-12 images. (**G**) Quantification of the spatial distribution of TAMs in 4T1. n = 3 mice, N = 9-12 images. (**H**) Quantification of the spatial distribution of CD11c+ cells in 4T1. n = 3 mice, N = 9-12 images. Data presented as average +/- standard error of the mean.


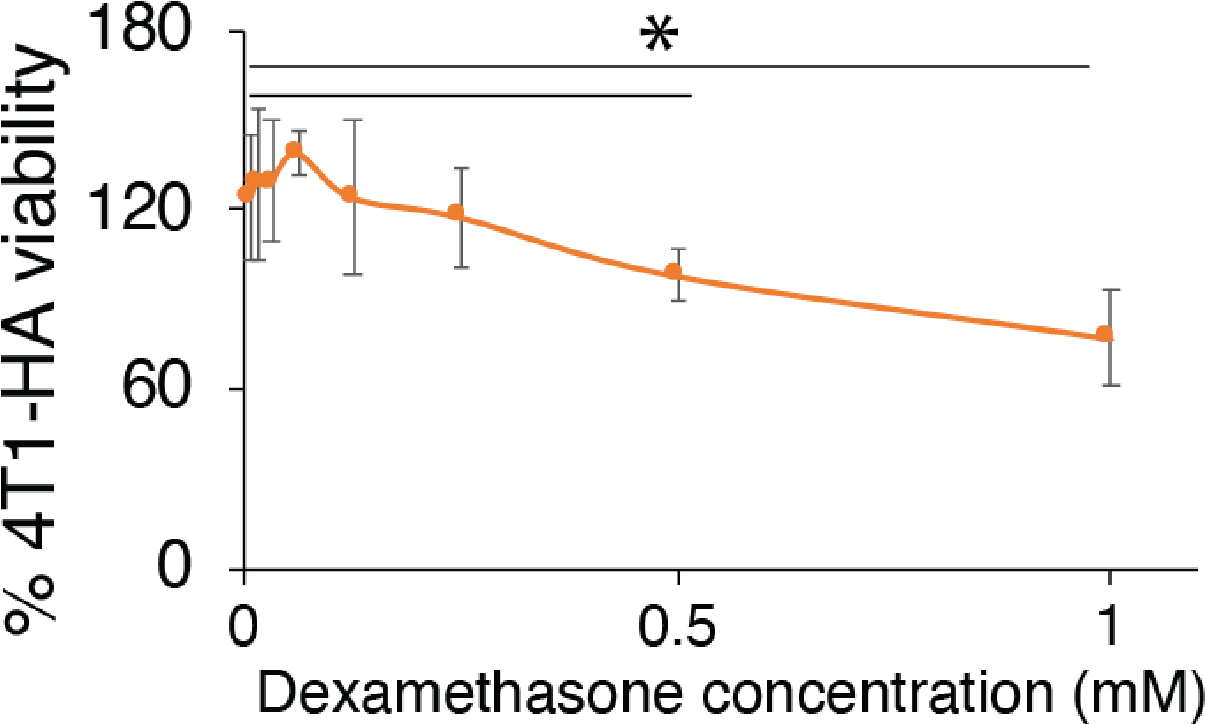


**Supplementary Figure 12. Dexamethasone is not toxic against 4T1-HA cells.** Cell viability plotted as a percentage of cell number before dexamethasone introduction versus dexamethasone concentration. Viability was not affected below 0.5 mM dexamethasone (P < 0.05, one-way ANOVA with Dunnett’s correction). n = 4 repetitions per concentration. Data plotted as mean +/- standard deviation.


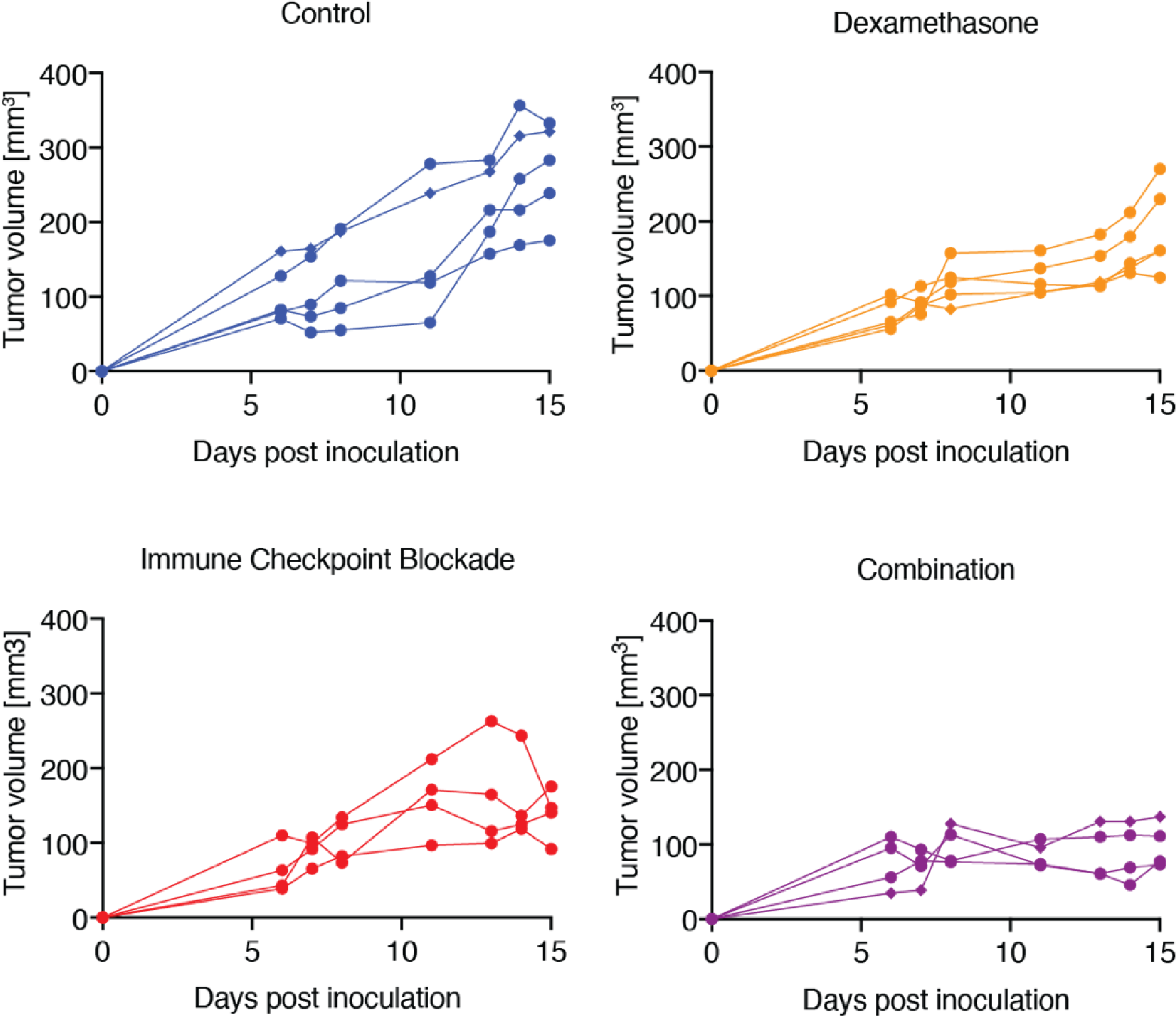


**Supplementary Figure 13. Tumor volume profiles of samples used for flow cytometry.** Tumor volumes of 4T1-HA tumors post inoculation and during treatment. n = 4-5 mice.

**
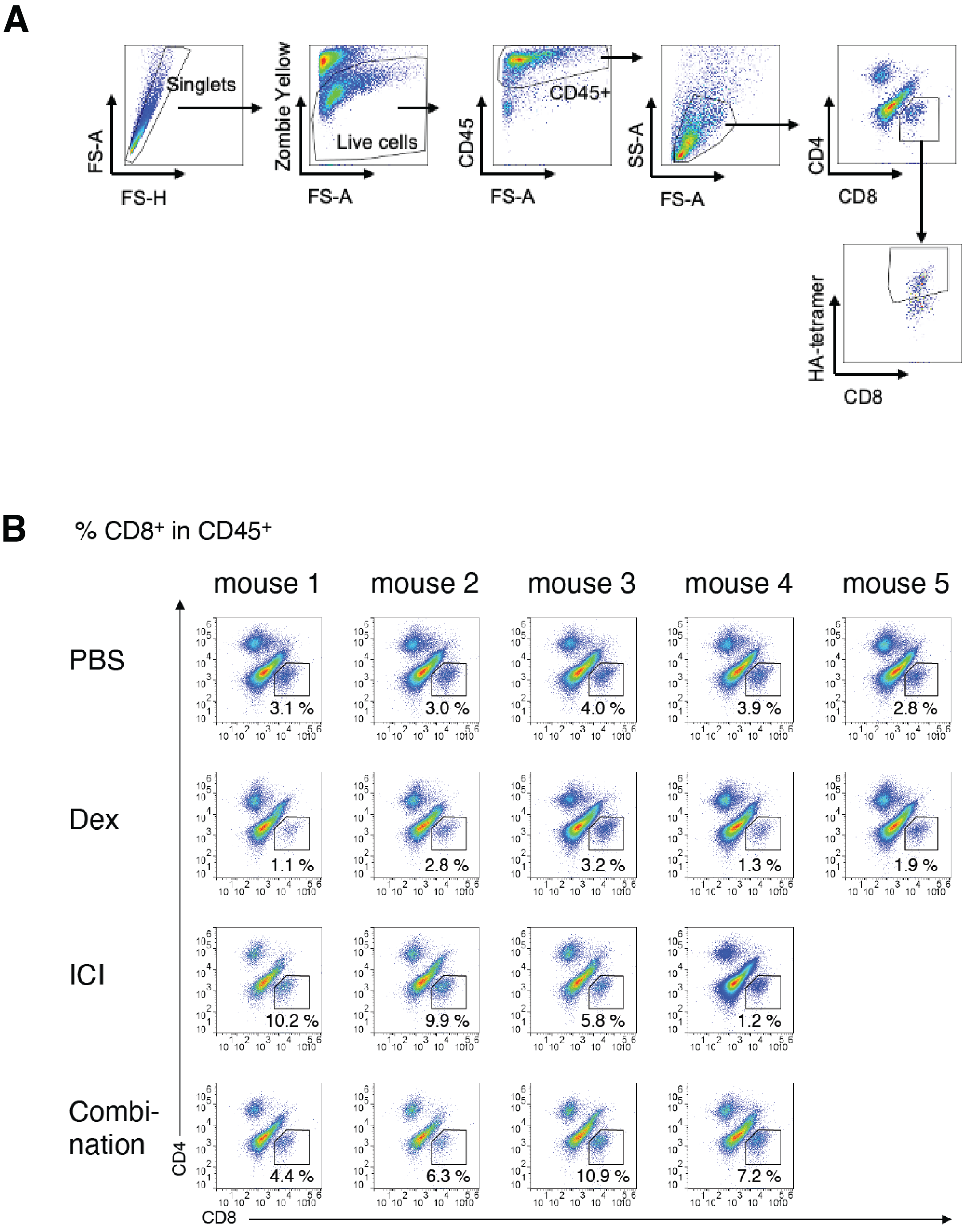
**

**Supplementary Figure 14. Flow cytometry gating of CD8^+^ lymphocytes.** (**A**) Flow cytometry gating of HA-specific CD8+ T cells in 4T1-HA experiments. (**B**) Tumor-infiltrating lymphocytes were detected as of CD8^+^ and CD4^+^ cells in the CD45^+^ population. Gatings are organized by treatment in rows and mouse in columns.


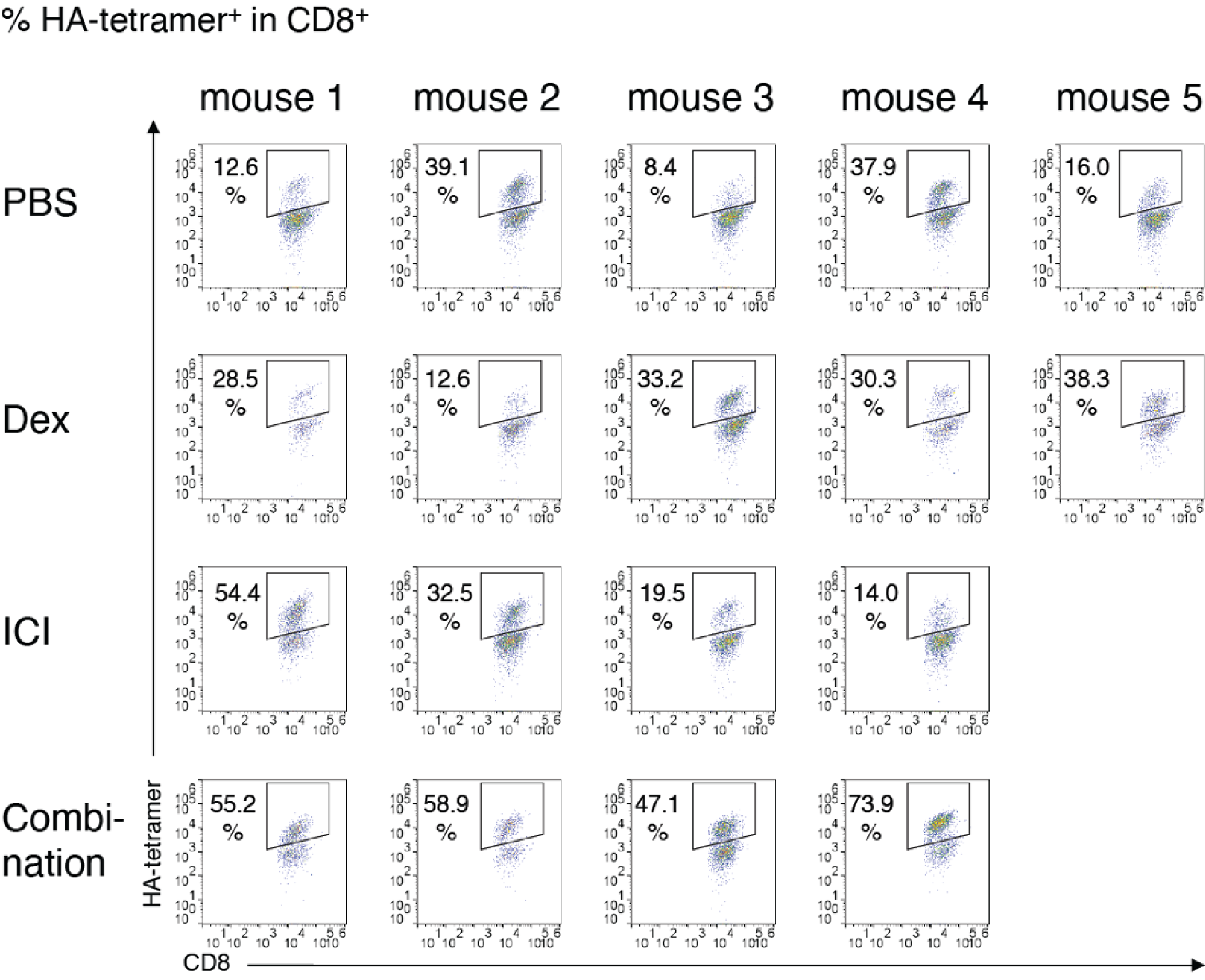


**Supplementary Figure 15*.* Flow cytometry gating of HA-tetramer^+^CD8^+^ antigen recognizing lymphocytes.** Tumor-infiltrating antigen recognizing lymphocytes were detected as HA-tetramer^+^ cells in the CD8^+^ population. Gatings are organized by treatment in rows and mouse in columns.

**
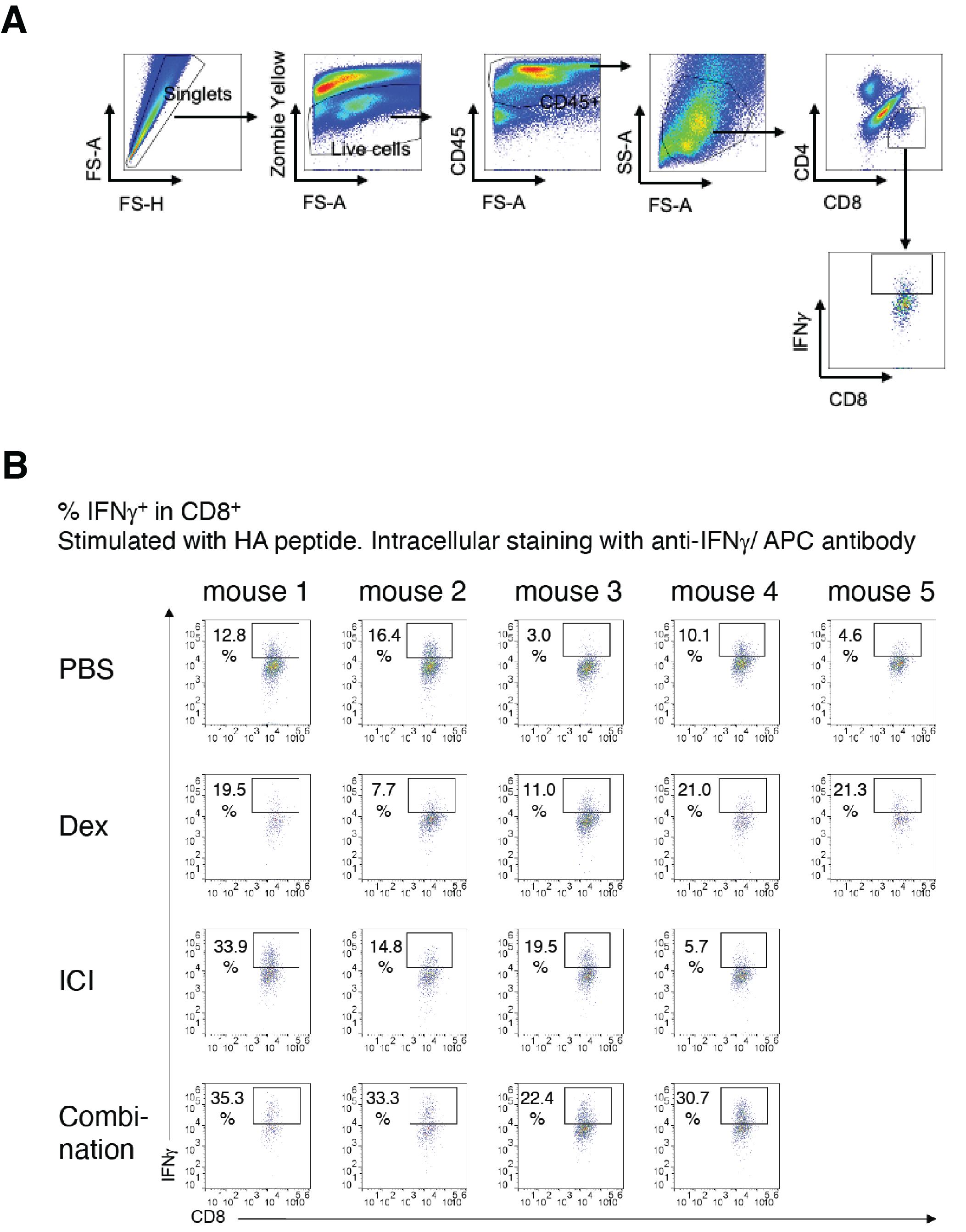
**

**Supplementary Figure 16. Flow cytometry gating of IFN**g **expressing CD8^+^ T cells after stimulation with HA peptide.** (**A**) Flow cytometry gating of HA-specific CD8+ T cells in 4T1-HA experiments. (**B**) Tumor-infiltrating CD8^+^ lymphocytes were stimulated with the HA peptide and staining of IFNg was performed. Gatings are organized by treatment in rows and mouse in columns.


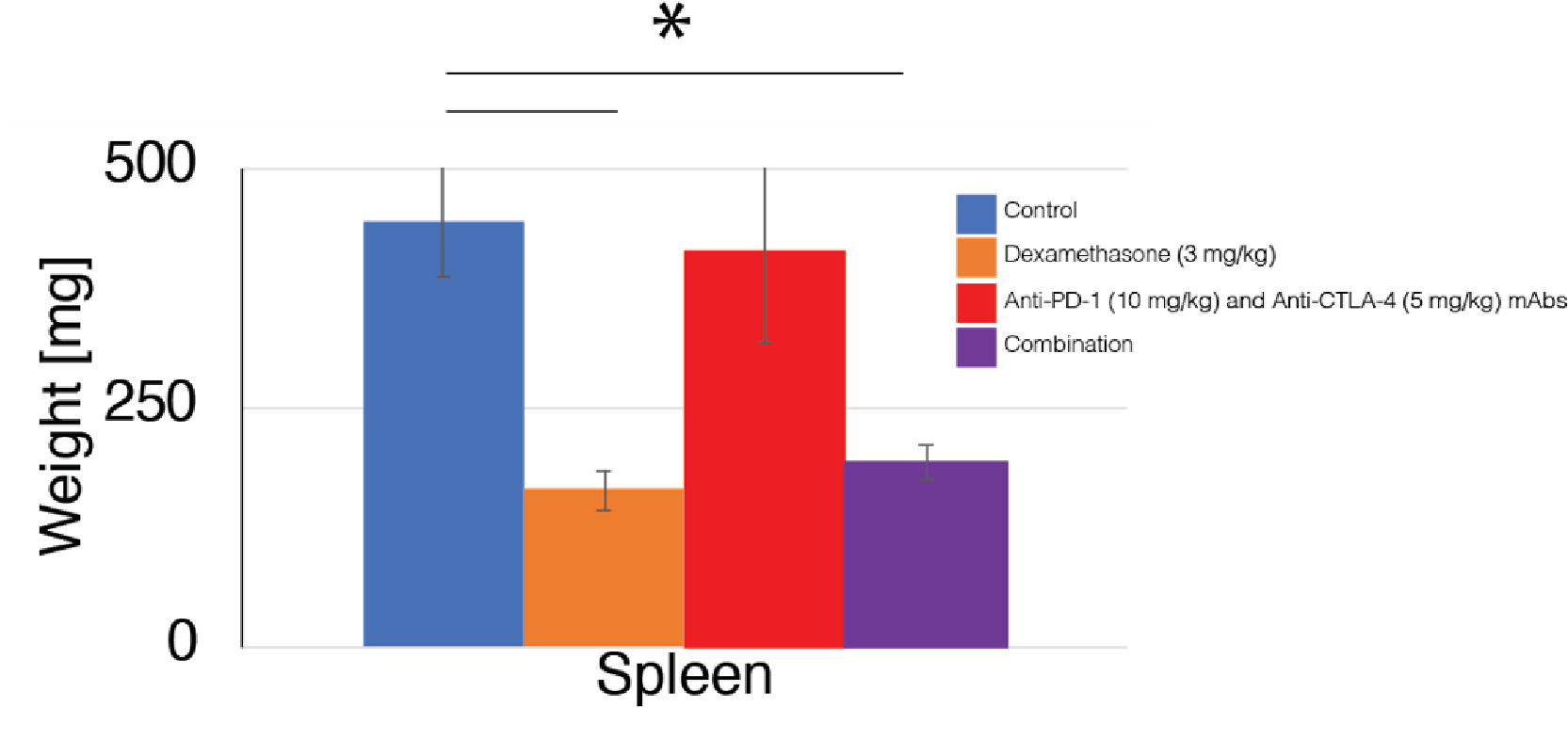


**Supplementary Figure 17. Dexamethasone decreased spleen weights on day 15 after inoculation of 4T1-HA cells.** Spleens were weighed, and dexamethasone monotherapy and the combination therapy groups had significantly lower weights (P < 0.05, one-way ANOVA with Dunnett’s correction). n = 4-5 mice. Data presented as average +/- standard error of the mean.


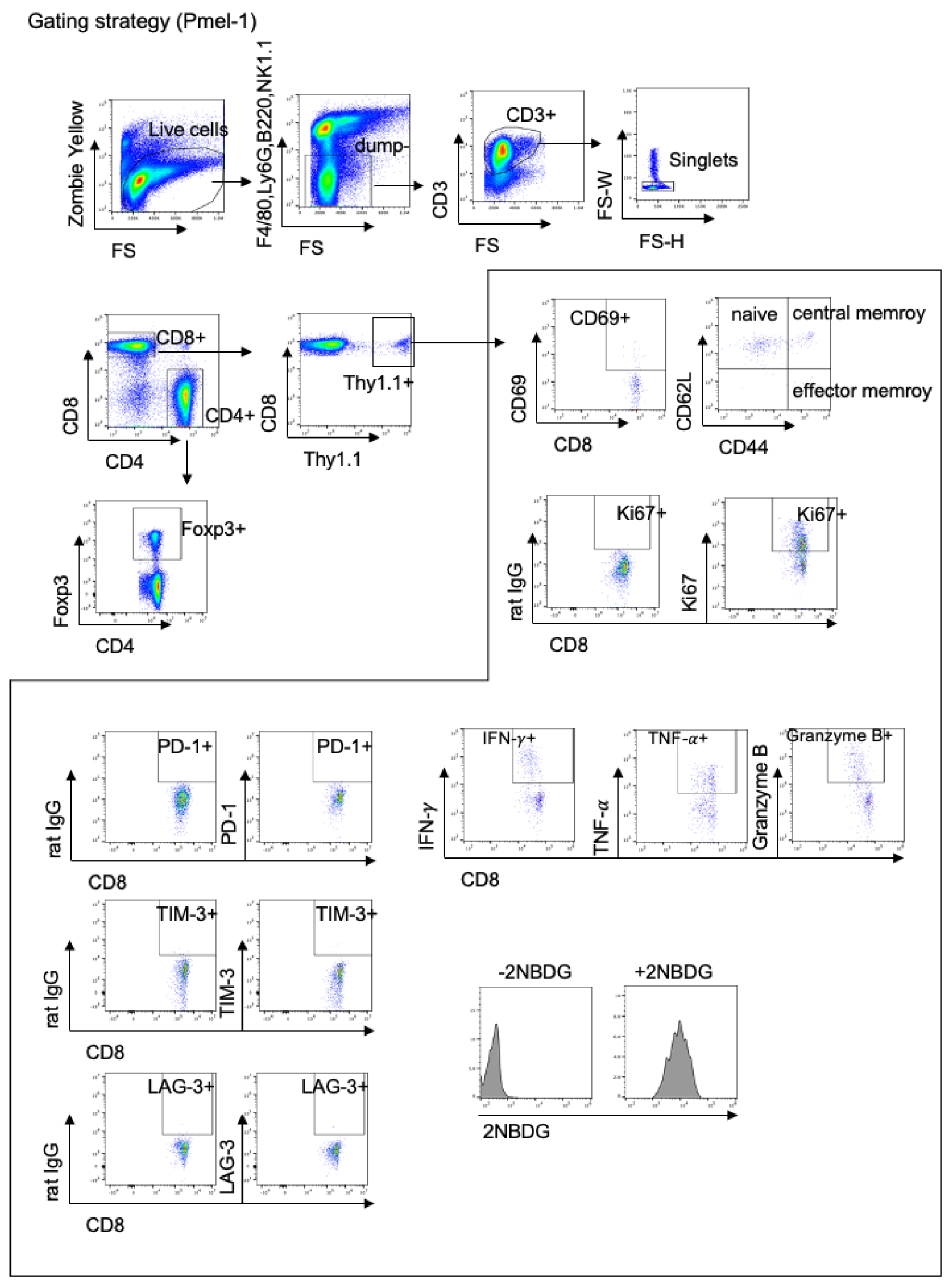


**Supplementary Figure 18. Flow cytometry gating of pmel-1 experiments.** Tumor infiltrating lymphocytes were detected as of CD3^+^CD8^+^ and CD3^+^CD4^+^ cells in the CD45^+^ leukocyte population after myeloid cell removal. Regulatory T cells and Thy1.1 cells were detected, and of the later, proliferating, activation, memory, immune checkpoints, intracellular cytokines, and glucose uptake were detected.


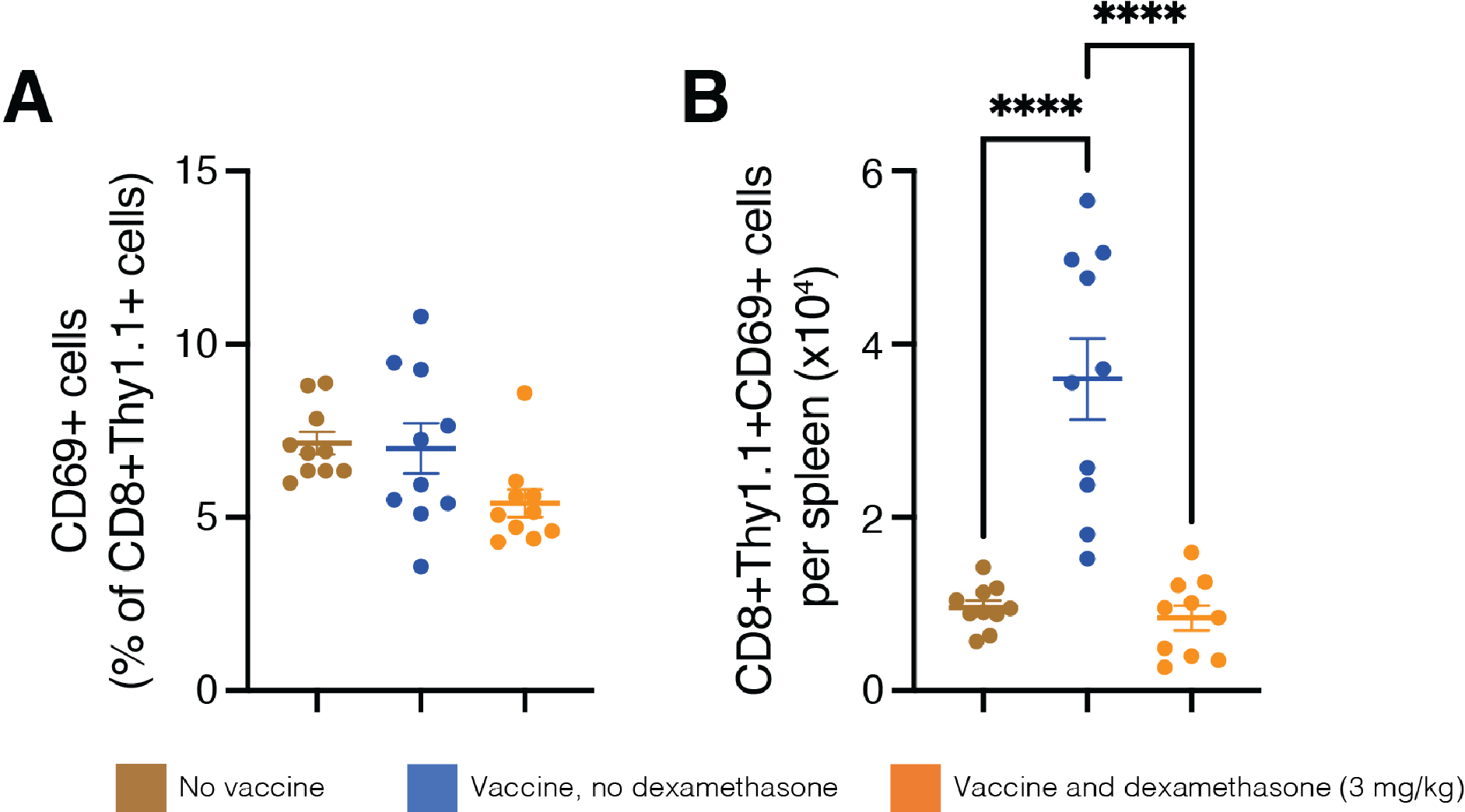


**Supplementary Figure 19. Dexamethasone reduces amount but not fraction of CD69+ cells in the spleens of antigen-naïve mice.** (**A**) CD69+ T cells as a percentage of tumor-specific (CD8+Thy1.1+) T cells. n = 10 mice per group. (**B**) The number of CD8+Thy1.1+ T cells per spleen. n = 10 mice per group. For all graphs, data plotted as average +/- standard error of the mean. Each dot represents one mouse. Statistical test by one-way ANOVA with Holm-Šídák’s correction (**** denotes P<0.0001, *** denotes P<0.001, ** denotes P<0.01, and * denotes *P* < 0.05).


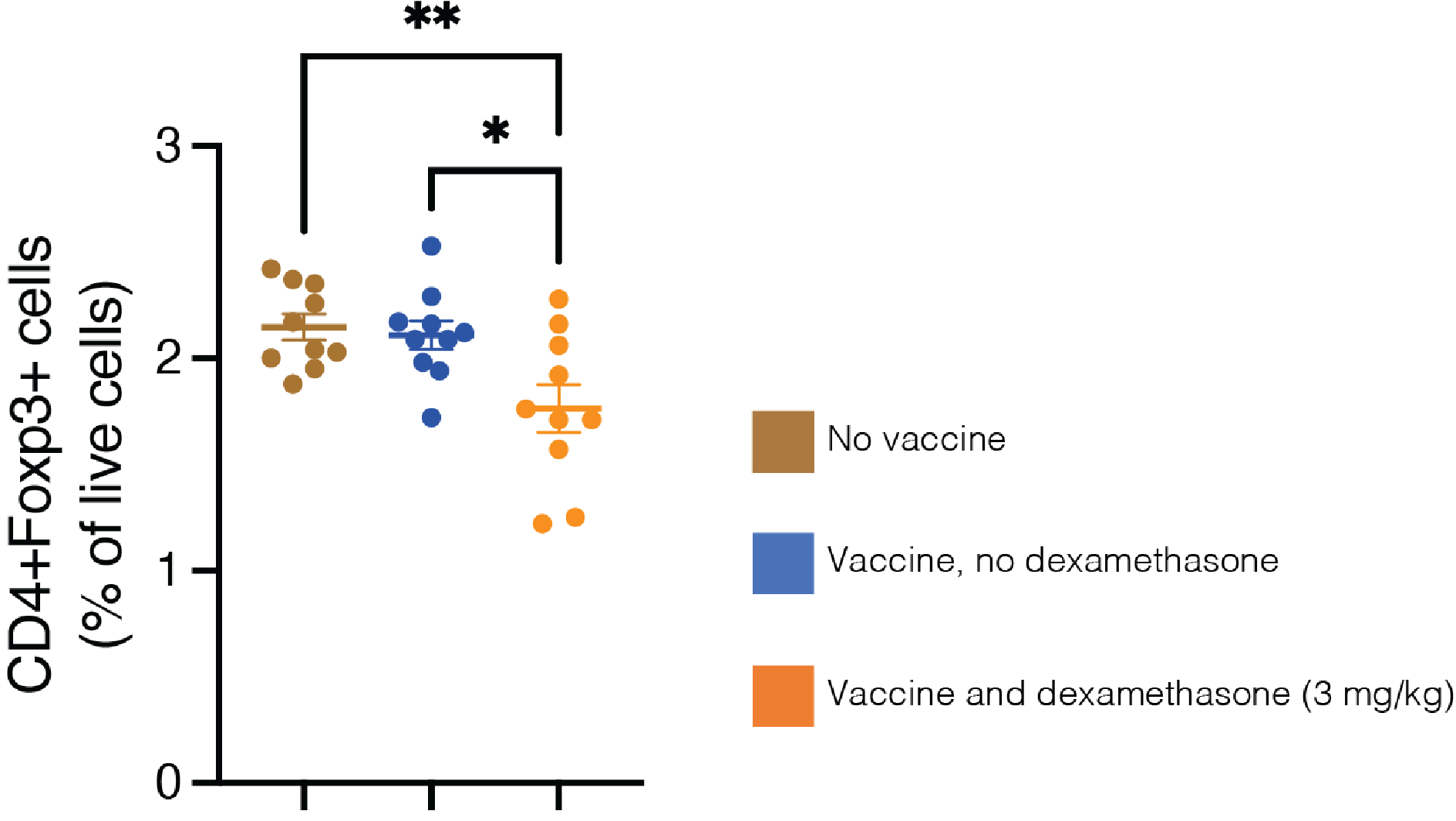


**Supplementary Figure 20. Dexamethasone reduces the fraction of CD4+Foxp3+ regulatory T cells in the spleens of antigen-naïve mice.** Regulatory T cells (CD4+Foxp3+) as a percentage of live cells. n = 10 mice per group. Data plotted as average +/- standard error of the mean. Each dot represents one mouse. Statistical test by one-way ANOVA with HolmŠídák’s correction (**** denotes P<0.0001, *** denotes P<0.001, ** denotes P<0.01, and * denotes *P* < 0.05).


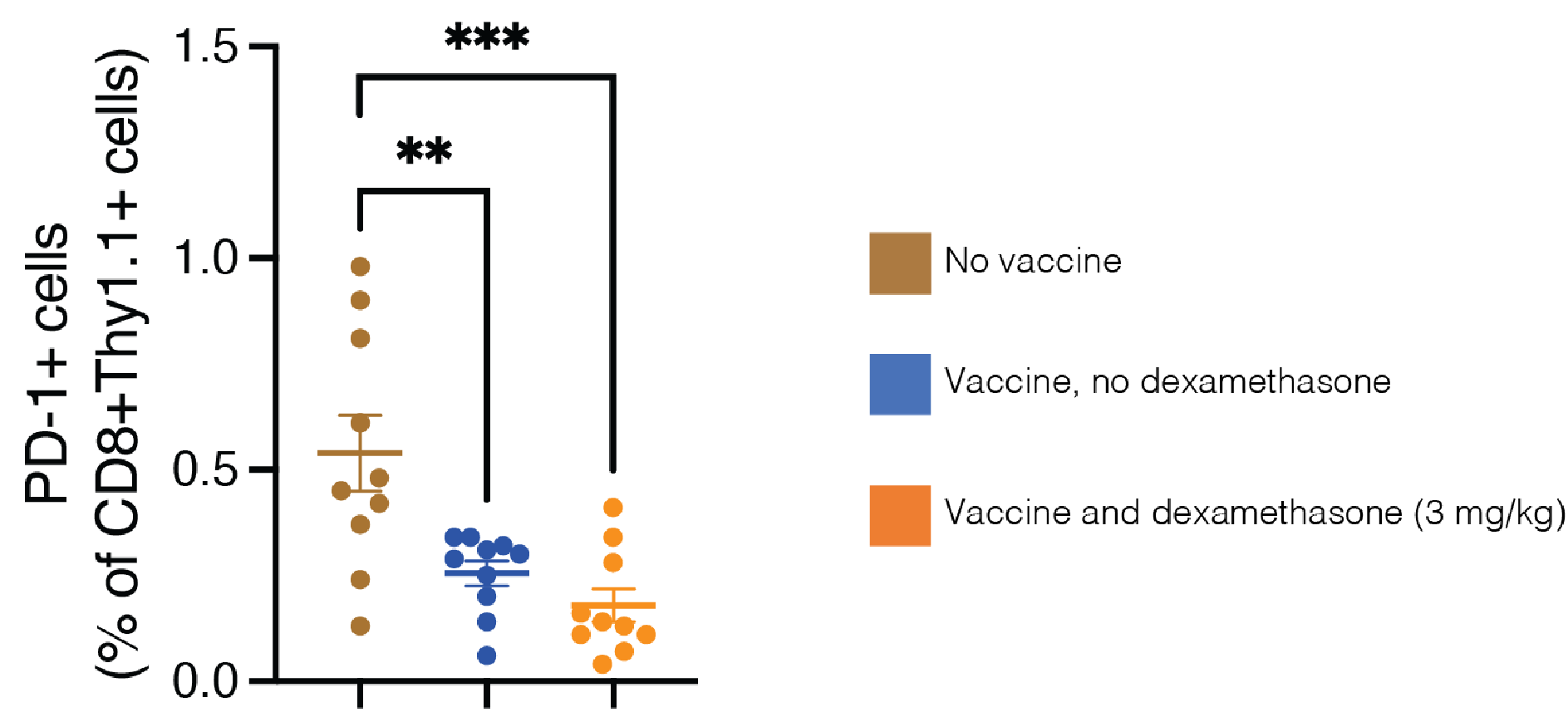


**Supplementary Figure 21. Few checkpoints are expressed on antigen-specific T cells in this antigen-naïve model, and dexamethasone does not affect the expression.** The fraction of PD-1-expressing tumor-specific (CD8+Thy1.1+) T cells. n = 10 mice per group. Data plotted as average +/- standard error of the mean. Each dot represents one mouse. Statistical test by one-way ANOVA with Holm-Šídák’s correction (**** denotes P<0.0001, *** denotes P<0.001, ** denotes P<0.01, and * denotes *P* < 0.05).


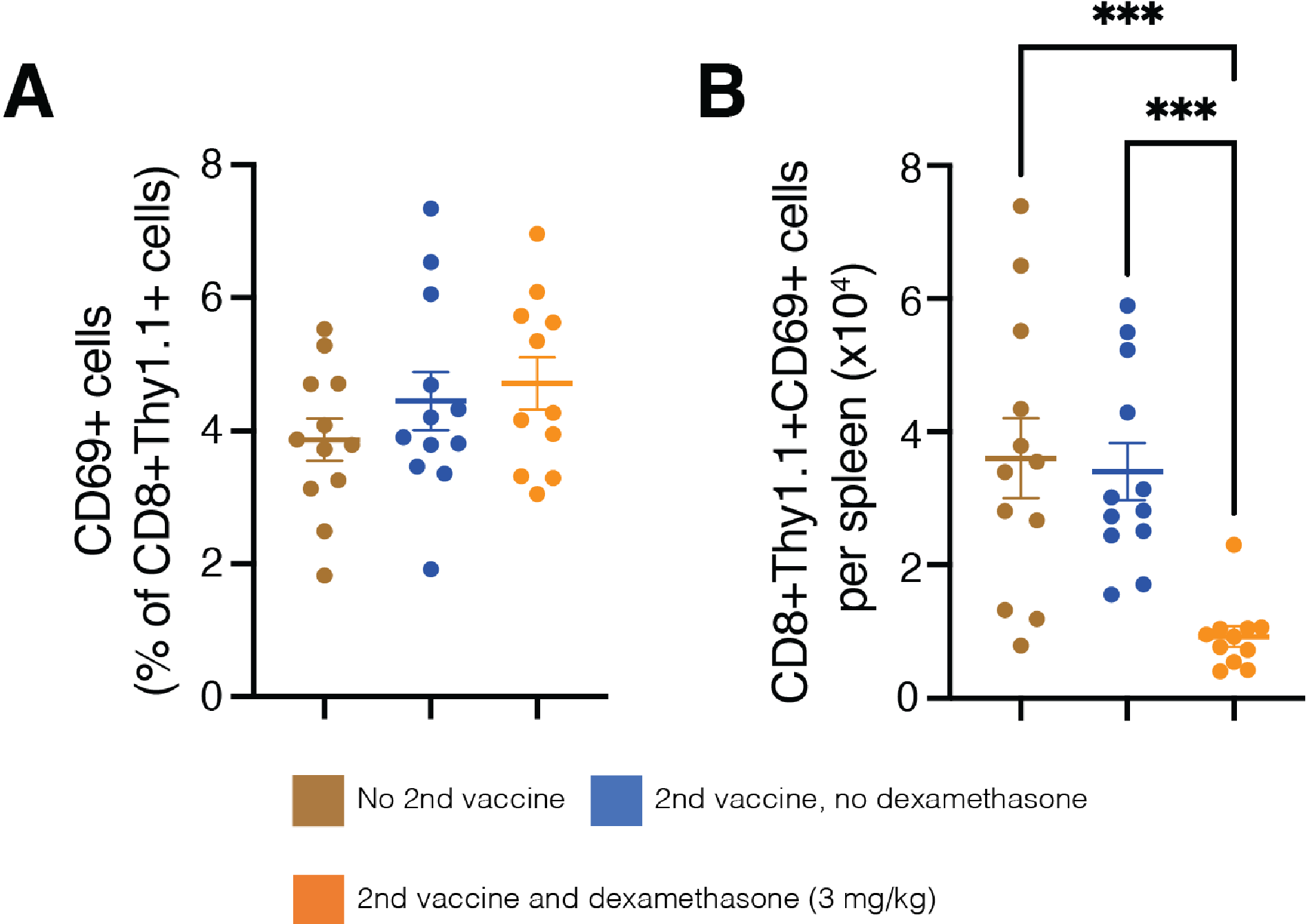


**Supplementary Figure 22. Dexamethasone reduces amount but not fraction of CD69+ cells in the spleens of antigen-experienced mice.** (**A**) CD69+ T cells as a percentage of tumor specific (CD8+Thy1.1+) T cells. n = 10 mice per group. (**B**) The number of CD8+Thy1.1+ T cells per spleen. n = 11-12 mice per group. For all graphs, data plotted as average +/- standard error of the mean. Each dot represents one mouse. Statistical test by one-way ANOVA with Holm-Šídák’s correction (**** denotes P<0.0001, *** denotes P<0.001, ** denotes P<0.01, and * denotes *P* < 0.05).


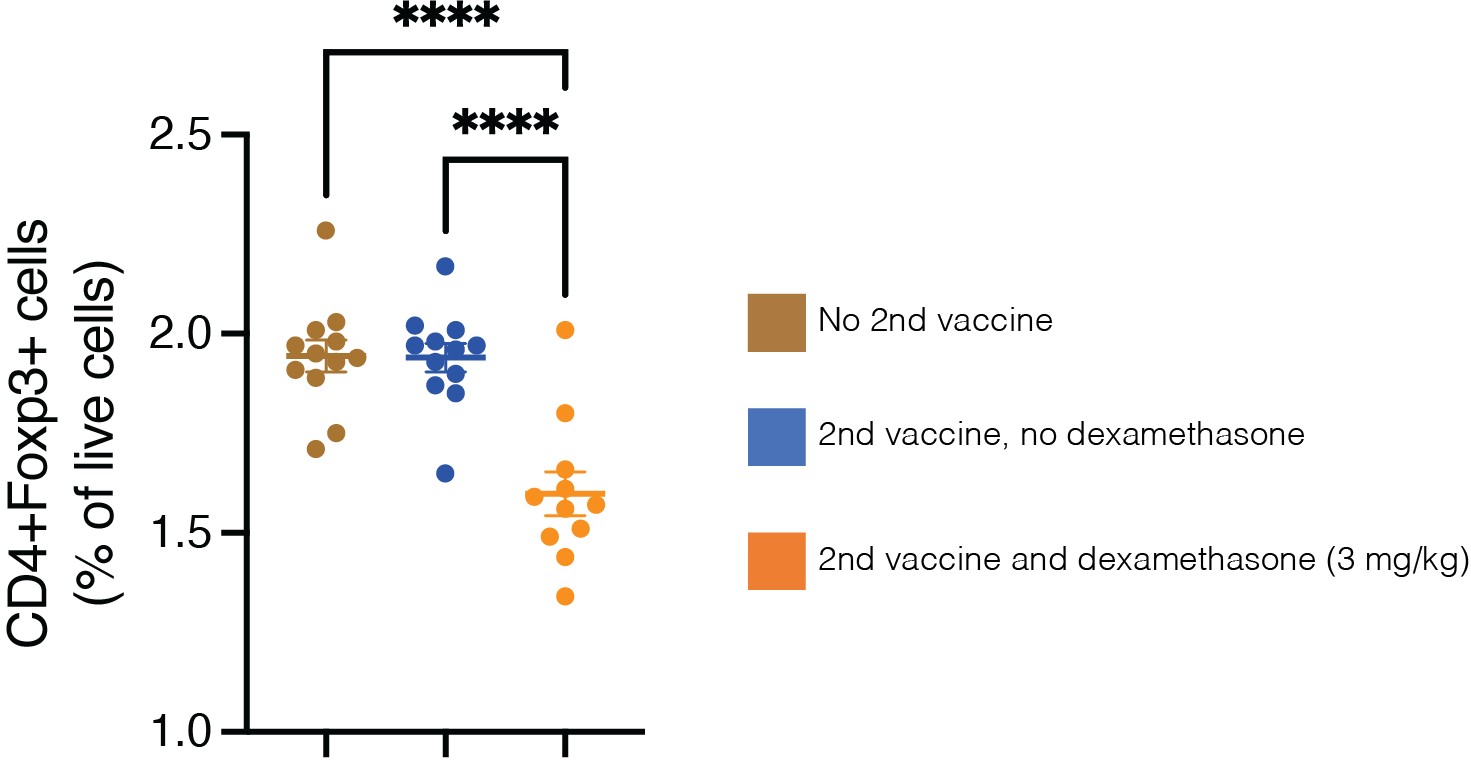


**Supplementary Figure 23. Dexamethasone reduces the fraction of CD4+Foxp3+ regulatory T cells in the spleens of antigen-experienced mice.** Regulatory T cells (CD4+Foxp3+) as a percentage of live cells. n = 11-12 mice per group. Data plotted as average +/- standard error of the mean. Each dot represents one mouse. Statistical test by one-way ANOVA with Holm-Šídák’s correction (**** denotes P<0.0001, *** denotes P<0.001, ** denotes P<0.01, and * denotes *P* < 0.05).


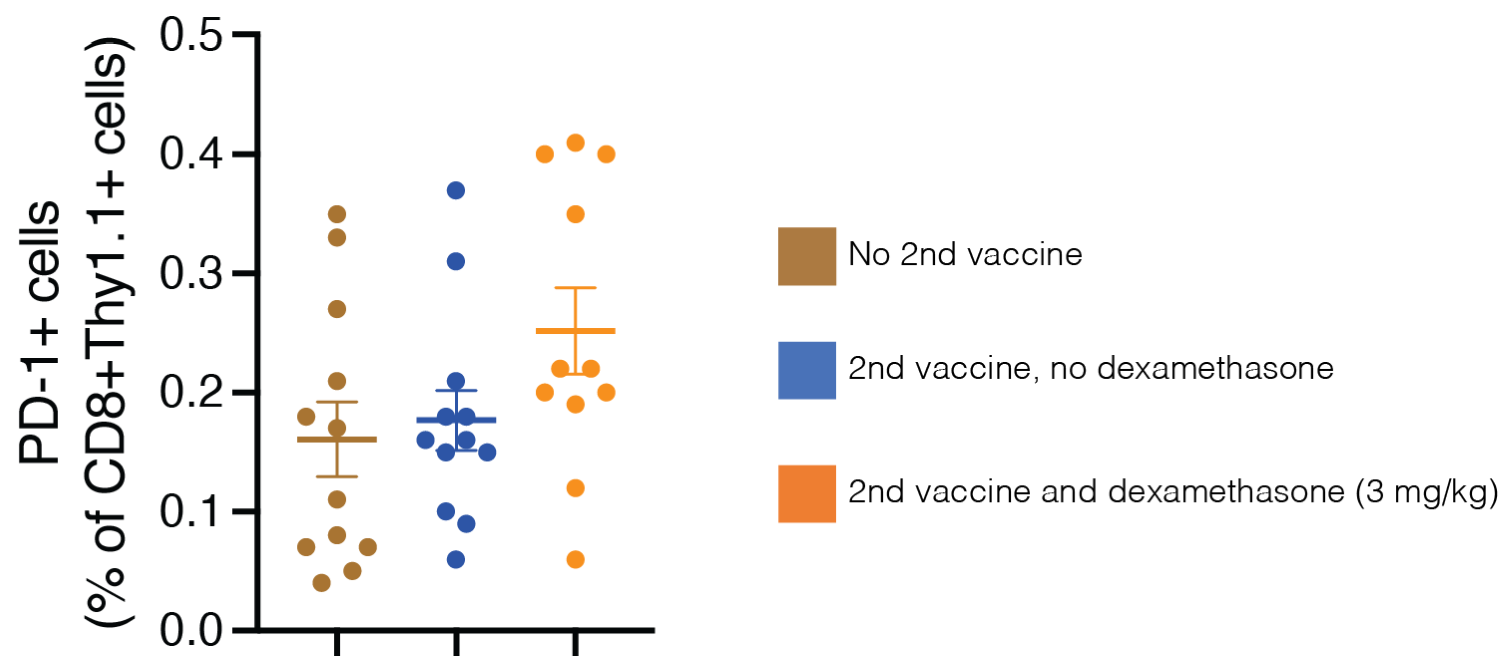


**Supplementary Figure 24. Few checkpoints are expressed on antigen-specific T cells in this antigen-experienced model, and dexamethasone does not affect the expression.** The fraction of PD-1-expressing tumor-specific (CD8+Thy1.1+) T cells. n = 11-12 mice per group. Data plotted as average +/- standard error of the mean. Each dot represents one mouse. Statistical test by one-way ANOVA with Holm-Šídák’s correction (**** denotes P<0.0001, *** denotes P<0.001, ** denotes P<0.01, and * denotes *P* < 0.05).


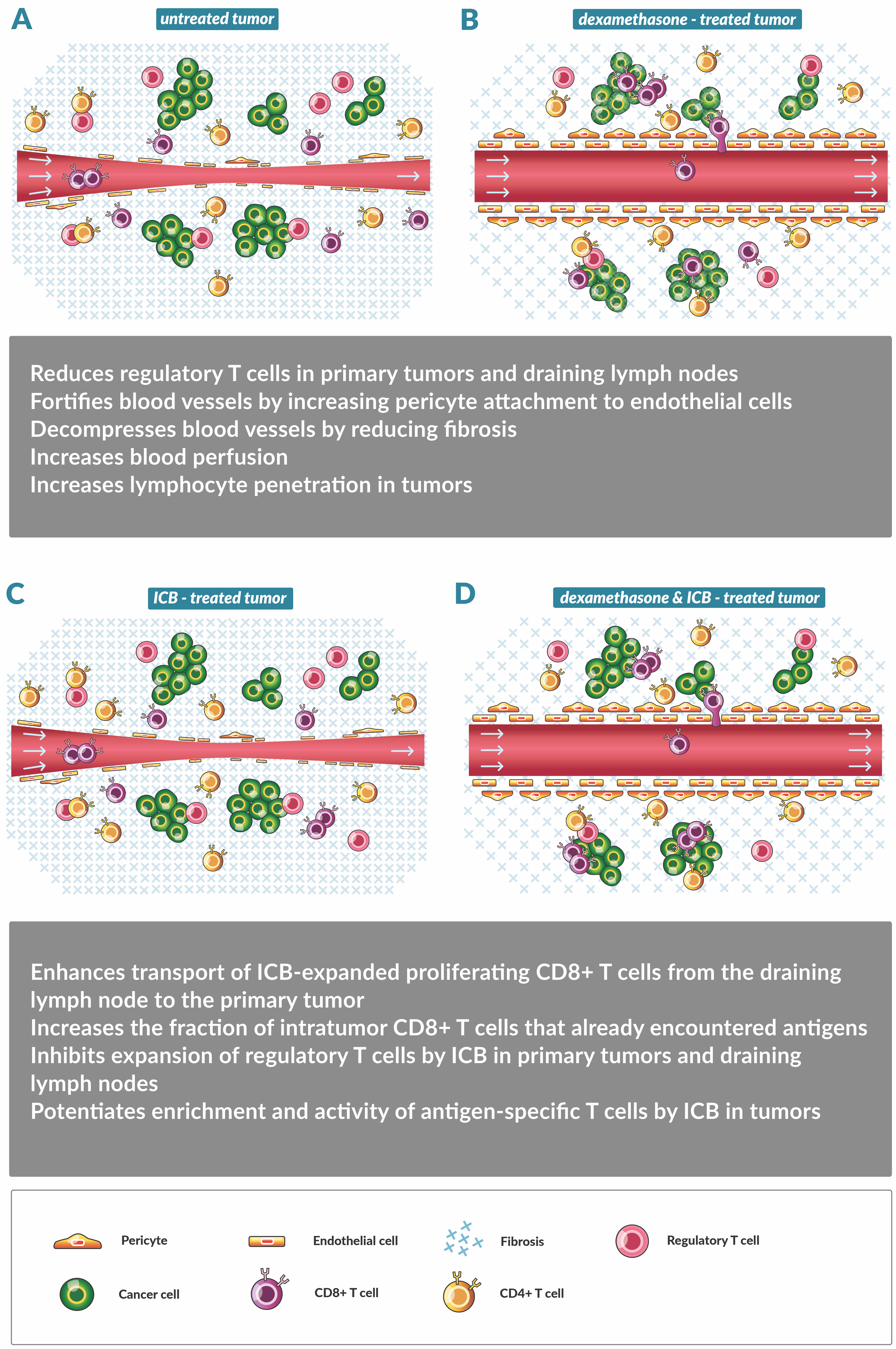


**Supplementary Figure 25. Graphical abstract.** (**A**) An illustrative schematic of a magnified, cross-section of a single blood vessel within the microenvironment of an untreated tumor. The blood vessel (red tube) is compressed in the center from fibrosis. The immature vessel also lacks a consistent endothelial cell layer and is not fortified with pericytes, so blood flow is restricted (gray arrows). Cancer cells, pericytes, endothelial cells, fibrosis, CD4+ T cells, and CD8+ T cells, and regulatory T cells (CD4+CD25+FOXP3+). CD8+ and other subsets of CD4+ T cells are sparse and unable to frequently reach the cancer cells in the tumor parenchyma. (**B**) A schematic of (**A**) after treatment with antiemesis dexamethasone. Dexamethasone depletes regulatory T cells in tumors and their draining lymph nodes. As dexamethasone reduces antiangiogenic signaling, the blood vessel matures with endothelial cells aligned and pericytes fortifying. Fibrosis is also reduced, resulting in decompressed vessels. Perfusion increases thereby enabling spatially homogenous lymphocyte penetration. Reduced angiogenic signalling could also promote the trafficking and transmigration of transiting immune cells such as CD8+ T cells. The lack of angiogenic and hypoxia signaling leads to fewer immunosuppressive cells. (**C**) A schematic of (**A**) after treatment with immune checkpoint blockers (ICB). ICB treatment increases the density of pro-and anti-tumor immune cells but does not increase the penetration of anti-tumor immune cells into the tumor. (**D**) A schematic of (**C**) after combined treatment of antiemesis dexamethasone with ICB. As in (**B**), fibrosis is reduced and the vessel is decompressed and mature. While dexamethasone directly depletes regulatory T cells, the associated reduction in immunosuppressive signaling in the tumor could cause a lower density of pro-tumor immune cells. The mature blood vessels and lack of fibrosis enable CD8+ T cells that were expanded by ICB enable proliferating CD8+ T cells to traffic from the draining lymph nodes to the tumor. As immune cells distribute throughout the tumor more evenly, more CD8+ T cells have encountered tumor antigens. While ICB expands regulatory T cells in primary tumors and the draining lymph nodes, dexamethasone counteracts this effect, leading to strong anti-tumor immune response. Perhaps because of reduced hypoxia and other immunosuppressive signaling, antigen-specific T cells are more active.

**Supplementary Movie 1. Vascular cast of control-treated 4T1 tumor.** High resolution 3D MR angiography (maximum intensity projection mapping) of subcutaneous transplanted 4T1 breast tumors of female BALB/c nude mice (circular protrusions).

**Supplementary Movie 2. Vascular cast of dexamethasone-treated 4T1 tumor.** High resolution 3D MR angiography (maximum intensity projection mapping) of subcutaneous transplanted 4T1 breast tumors of female BALB/c nude mice (circular protrusions) after treatment with the TME-normalizing dose of dexamethasone (3 mg/kg).

**References**

1. J. D. Martin *et al.*, Dexamethasone Increases Cisplatin-Loaded Nanocarrier Delivery and Efficacy in Metastatic Breast Cancer by Normalizing the Tumor Microenvironment. *ACS Nano* **13**, 6396-6408 (2019).
2. P. E. Kristjansen, Y. Boucher, R. K. Jain, Dexamethasone reduces the interstitial fluid pressure in a human colon adenocarcinoma xenograft. *Cancer research* **53**, 4764-4766 (1993).
3. K. C. Arbour *et al.*, Impact of Baseline Steroids on Efficacy of Programmed Cell Death-1 and Programmed Death-Ligand 1 Blockade in Patients With Non–Small-Cell Lung Cancer. *Journal of Clinical Oncology*, JCO. 2018.2079. 0006 (2018).
4. V. Subbiah *et al.*, Phase 1b/2 Trial of NC-6004 (Nanoparticle Cisplatin) Plus Gemcitabine in Patients with Advanced Solid Tumors. *Clinical Cancer Research* **24**, 43-51 (2017).
5. C. J. Vecht, A. Hovestadt, H. Verbiest, J. Van Vliet, W. Van Putten, Dose‐effect relationship of dexamethasone on Karnofsky performance in metastatic brain tumors A randomized study of doses of 4, 8, and 16 mg per day. *Neurology* **44**, 675-675 (1994).
